# Common Factors in Neurodegeneration: A Meta-Study Revealing Shared Patterns on a Multi-Omics Scale

**DOI:** 10.3390/cells9122642

**Published:** 2020-12-08

**Authors:** Nicolas Ruffini, Susanne Klingenberg, Susann Schweiger, Susanne Gerber

**Affiliations:** 1 Institute for Human Genetics, University Medical Center, Johannes Gutenberg University, 55131 Mainz, Germany; Nicolas.Ruffini@lir-mainz.de (N.R.); klingens@uni-mainz.de (S.K.); schweigs@uni-mainz.de (S.S.); 2 Leibniz Institute for Resilience Research, Leibniz Association, Wallstraße 7, 55122 Mainz, Germany

**Keywords:** multi-omics, Alzheimer’s disease, Parkinson’s disease, Huntington’s disease, amyotrophic lateral sclerosis, neurodegeneration

## Abstract

Neurodegenerative diseases such as Alzheimer’s disease (AD), Parkinson’s disease (PD), Huntington’s disease (HD), and amyotrophic lateral sclerosis (ALS) are heterogeneous, progressive diseases with frequently overlapping symptoms characterized by a loss of neurons. Studies have suggested relations between neurodegenerative diseases for many years (e.g., regarding the aggregation of toxic proteins or triggering endogenous cell death pathways). We gathered publicly available genomic, transcriptomic, and proteomic data from 177 studies and more than one million patients to detect shared genetic patterns between the neurodegenerative diseases on three analyzed omics-layers. The results show a remarkably high number of shared differentially expressed genes between the transcriptomic and proteomic levels for all conditions, while showing a significant relation between genomic and proteomic data between AD and PD and AD and ALS. We identified a set of 139 genes being differentially expressed in several transcriptomic experiments of all four diseases. These 139 genes showed overrepresented gene ontology (GO) Terms involved in the development of neurodegeneration, such as response to heat and hypoxia, positive regulation of cytokines and angiogenesis, and RNA catabolic process. Furthermore, the four analyzed neurodegenerative diseases (NDDs) were clustered by their mean direction of regulation throughout all transcriptomic studies for this set of 139 genes, with the closest relation regarding this common gene set seen between AD and HD. GO-Term and pathway analysis of the proteomic overlap led to biological processes (BPs), related to protein folding and humoral immune response. Taken together, we could confirm the existence of many relations between Alzheimer’s disease, Parkinson’s disease, Huntington’s disease, and amyotrophic lateral sclerosis on transcriptomic and proteomic levels by analyzing the pathways and GO-Terms arising in these intersections. The significance of the connection and the striking relation of the results to processes leading to neurodegeneration between the transcriptomic and proteomic data for all four analyzed neurodegenerative diseases showed that exploring many studies simultaneously, including multiple omics-layers of different neurodegenerative diseases simultaneously, holds new relevant insights that do not emerge from analyzing these data separately. Furthermore, the results shed light on processes like the humoral immune response that have previously been described only for certain diseases. Our data therefore suggest human patients with neurodegenerative diseases should be addressed as complex biological systems by integrating multiple underlying data sources.

## 1. Introduction

Neurodegenerative diseases (NDDs), including Alzheimer’s disease, Parkinson’s disease, amyotrophic lateral sclerosis, and Huntington’s disease, are heterogeneous, progressive diseases characterized by a loss of neurons, an accumulation of aggregated and misfolded proteins [1,2,3,4], cognitive decline and locomotive dysfunction [5,6,7]. Despite decades of research and considerable progress in identifying risk genes, potent biomarkers, and environmental risk factors, this progression cannot be impeded. As details regarding the various (patho-)physiological processes associated with neurodegenerative diseases remain unclear, the diseases are still incurable. At the same time, it has become generally accepted that the underlying mechanisms are polyfactorial and depend on the complex interplay of multiple (partly unknown) genetic and non-genetic variables [8,9,10,11,12,13,14].

**Figure 1:**
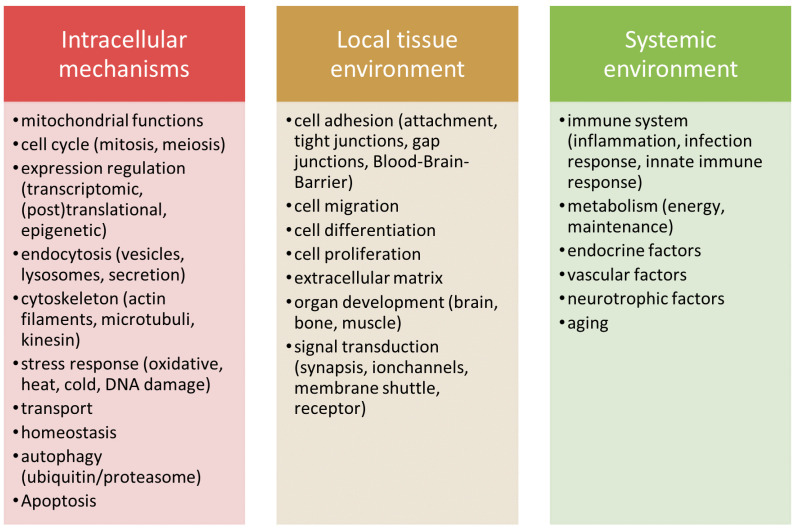
Classification of candidate pathways contributing to neurodegeneration into three groups according to their cellular mechanisms or their primary site of action. The categorization is based on Ramanan’s pathways to neurodegeneration [15] and aims to help in classifying the current knowledge surrounding neurodegeneration.

Influences on the development of NDDs can be classified into broader functional groups based on their primary site or mode of action into intracellular mechanisms, local tissue environment influences, and systemic influences [15]. These pathways and mechanisms are highly related and can have overlapping or interacting components that can collectively modulate neurodegenerative processes. We slightly adapted this categorization based on Ramanan [15], and depict the processes associated with each of the three categories in Figure 1. Candidate pathways influencing the balance of neuronal survival and degeneration within the cell are misguided apoptosis and autophagy [16,17], dysfunction in mitochondria [18,19,20], various forms of cell stress [15,21,22], defective cytoskeletal proteins and impaired protein expression regulation [23,24,25,26]. Within the local tissue environment, impaired cell adhesion pathways lead to limited neurotransmission and cell proliferation, a permeable blood–brain barrier (BBB), and dysfunctional extracellular matrices (ECMs) [27,28,29]. Excessive immune response and inflammation [30,31,32,33] and a dysregulated lipid and sugar metabolism lead to disturbances in the whole systemic environment [34,35,36].

By far the most prevalent of NDDs, Alzheimer’s disease (AD) is an inexorably progressive brain disorder that affects higher cognitive functions [37,38,39]. The accumulation of abnormally folded extracellular β-amyloid (senile plaques) and intracellular phosphorylated tau (neurofibrillary tangles) proteins are the distinctive pathological hallmarks of the disease that might trigger synaptopathies, glial inflammation and eventually neuronal death in the cerebral cortex, subcortical regions, temporal and parietal lobes and cingulate gyrus [40,41,42] and even effects the gut microbiome [43].

Parkinson’s disease (PD) is the second most common neurodegenerative disorder, mainly affecting the motor system [44,45]. The aggregation of α-synuclein into Lewy bodies and Lewy neurites, primarily in the substantia nigra pars compacta, and the resulting loss of dopaminergic neurons leads to distinctive symptoms including resting tremors, bradykinesia, stooped posture and, in some cases, dementia [46,47,48].

Huntington’s disease (HD) is a progressive neurodegenerative disease that can lead to chorea, cognitive decline, psychiatric disorders and depression [49,50]. It manifests pathologically with the significant loss of the striatum’s GABAergic medium-sized spiny neurons [51,52] due to the intracellular accumulation of misfolded Huntingtin protein [53,54]. While both, familial and sporadic forms of AD and PD exist, HD is an autosomal dominant neurodegenerative disease caused by the expansion of a CAG repeat in the exon 1 of the huntingtin gene translating into a polyglutamine (polyQ) expansion in the N-terminus of the Huntingtin protein [54,55,56].

Amyotrophic lateral sclerosis (ALS) is a neurodegenerative disease affecting both the upper and lower motor neurons [57,58,59], and is characterized by progressive muscular paralysis reflecting the degeneration of motor neurons in the primary motor cortex, corticospinal tracts, brainstem and spinal cord [60,61,62]. Paralysis is progressive and leads to death due to respiratory failure within 2–5 years [57,58]. Most ALS cases are sporadic, but 5–10% of cases are familial with mutations of the *SOD1* and *TARDBP* (TDP-43) genes [59,63]. Cellular aggregates, including FUS, SOD1, TDP-43, OPTN, UBQLN2, and the translational product of intronic repeats in the gene *C9ORF72* are found both in the sporadic and the familial form [64].

**Figure 2:**
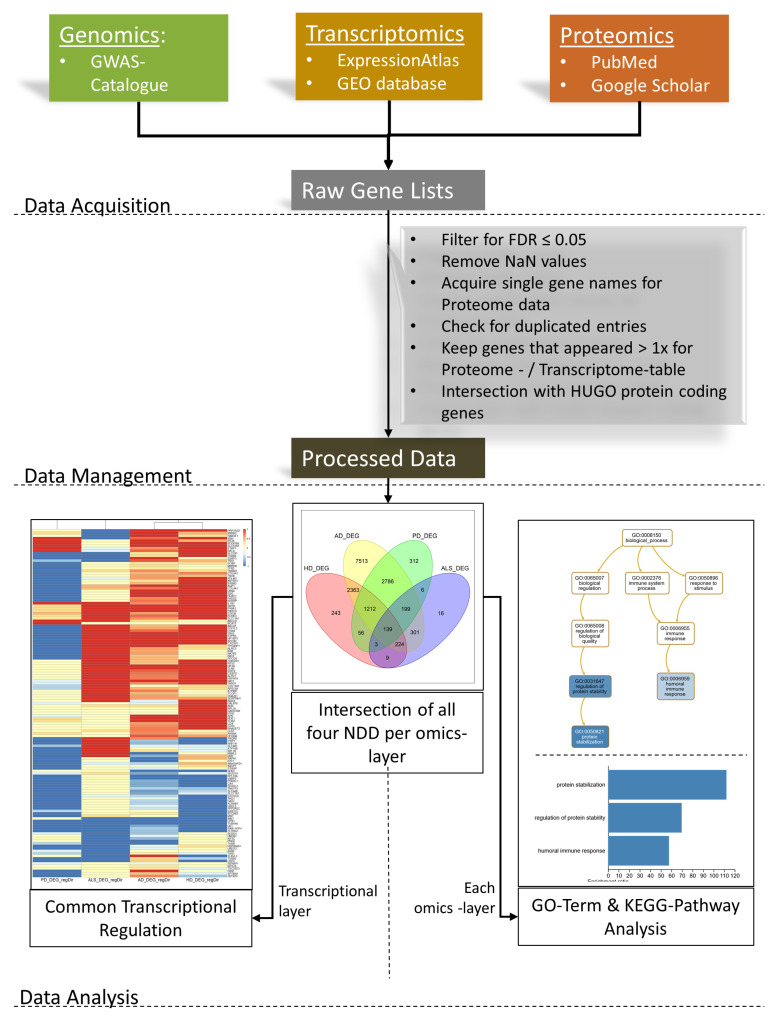
Workflow Overview: Data acquisition was performed using the genome-wide association studies (GWAS) Catalog for genomic data, the European Bioinformatics Institute (EMBL-EBI) Expression Atlas and the Gene Expression Omnibus database for transcriptomic data, and literature research in PubMed and Google Scholar for proteomic data. After filtering these raw data tables and applying some data transformation, the processed data were used for the data analysis. For every omics layer, the intersections of all four analyzed NDDs were visualized as Venn diagrams. Common transcriptional patterns were searched with a hierarchical clustering approach and visualized as a heatmap showing the mean transcriptional direction of regulation per gene, and a dendrogram showing the clustering results. Finally, each set of genes after the intersections was used for the Kyoto Encyclopedia for Genes and Genomes (KEGG) pathway and GO-Term analyses.

The described overlap of phenotypic traits of the NDD suggests common pathogenic mechanisms underlying distinct NDDs. Compared to studying individual diseases separately, identifying and analyzing the common dysfunctional proteins and dysregulated diseases’ pathways might elucidate fundamental insights into their pathogenic process [65]. It was previously shown that there is nearly no overlap between AD, PD, and ALS on genomic data and some shared pathways for AD, PD, ALS, and HD in transcriptomic data [66], but proteomic data and the latest entries in the databases have not been considered. Besides looking for overlapping genes between the different NDDs or omics layers, we also analyzed whether this number is sufficiently high to claim a significant relationship between NDDs or omics layers. An overview of the methodologic procedure is given in Figure 2. By investigating 177 studies in total, this meta-study was able to detect stable signals that arise mainly in late-stage NDDs across tissues, methods and omics layers, which could help unravel patterns across neurodegenerative diseases. Such findings could contribute to a better understanding of the underlying neurodegeneration process and might also have pharmacological relevance for various neurodegenerative diseases.

## 2. Materials and Methods

### 2.1. Data Acquisition/Literature Research

#### 2.1.1. Genome

The genome-wide association studies (GWAS) Catalog data for Alzheimer’s disease (AD), Parkinson’s disease (PD), amyotrophic lateral sclerosis (ALS) and Huntington’s disease (HD) were downloaded on 28 April 2020. The GWAS Catalog contains single nucleotide polymorphism (SNP) data of GWAS studies for SNPs showing a statistical significance of SNP-trait *p*-value < 1 × 10^−5^. in the overall population. For every SNP, data such as *p*-value, upstream gene(s), mapped gene, reported gene(s) and many more are stored. We focused on the genes given as “Reported Gene(s)” in the four examined diseases’ full data tables for our analysis. The experimental factor ontology (EFO) numbers for the exact search pattern were EFO_0000249 (Alzheimer’s disease), EFO_0002508 (Parkinson’s disease), Orphanet_399 (Huntington’s disease), and EFO_0000253 (Amyotrophic lateral sclerosis). A table containing all studies’ names and the number of investigated samples for each disease is appended in the Supplementary Table S1. In total, 116 studies with genomic data were used for the analyses [67,68,69,70,71,72,73,74,75,76,77,78,79,80,81,82,83,84,85,86,87,88,89,90,91,92,93,94,95,96,97,98,99,100,101,102,103,104,105,106,107,108,109,110,111,112,113,114,115,116,117,118,119,120,121,122,123,124,125,126,127,128,129,130,131,132,133,134,135,136,137,138,139,140,141,142,143,144,145,146,147,148,149,150,151,152,153,154,155,156,157,158,159,160,161,162,163,164,165,166,167,168,169,170,171,172,173,174,175,176,177,178,179,180,181,182], seven of which contained data of more than one of these NDDs.

#### 2.1.2. Transcriptome

We browsed the Gene Expression Omnibus (GEO) [183] and the Expression Atlas [184] databases. The GEO is a public data repository in which microarray and RNA-seq datasets can be found. The keywords for the GEO database were <name of disease> AND (“microarray” OR “RNAseq”) AND “human”. The latest literature research was done in July 2020. The Expression Atlas is a service of the European Bioinformatics Institute (EMBL-EBI) and provides re-analyzed and manually curated data of more than 3000 experiments. It was used in release 35 (May 2020, https://www.ebi.ac.uk/gxa/home) and scanned for Alzheimer, Parkinson, Huntington and amyotrophic lateral sclerosis, using the filter “*Homo sapiens*” in the section Differential Experiments.

An overview of all of the studies used to gather the transcriptomic data is provided in Table 1. In the Supplementary materials (Table S2), a table is provided showing each study’s information and a table of the proportion of all used tissues and severity states per disease (Supplementary File S4). Of the studies used, 55% utilized microarray experiments, 40% used RNA sequencing and 5% were based on single cell RNA sequencing experiments. In total, transcriptomic data of 39 studies was acquired [185,186,187,188,189,190,191,192,193,194,195,196,197,198,199,200,201,202,203,204,205,206,207,208,209,210,211,212,213,214,215,216,217,218,219,220,221,222,223].

#### 2.1.3. Proteome

We browsed publications from the last 10 years in PubMed and Google Scholar with the keywords: (“neurodegenerative diseases” OR “Alzheimer* disease” OR “Parkinson* disease” OR “Huntington* disease” OR “Amyotrophic Lateral Sclerosis”) AND (proteomics OR “quantitative proteomics” OR “differentially expressed proteins” OR biomarkers) AND human NOT mice. An overview statistic regarding the number of samples and studies for the proteomic data is given in Table 2. A table showing each study’s information is provided in the Supplementary materials (Table S3), as well as a table of the proportion of all used tissues and severity states per disease (Supplementary File S4). In total, 22 studies were used for proteomic data acquisition [36,50,190,193,224,225,226,227,228,229,230,231,232,233,234,235,236,237,238,239,240].

More than 90% of the non-control patients in the proteomic data and 84% in the transcriptomic data were classified with a moderate or severe disease state. Furthermore, 63% of the transcriptomic data experiments and 67% of the proteomic data experiments were conducted with brain material. The remaining experiments were conducted with blood, spinal cord, cerebral spinal fluid or induced pluripotent stem-cells. A detailed table can be found in the Supplementary materials (Table S3). Studies that turned out to show no single gene with a false discovery ratio < 0.05 were not considered for our statistics in Table 1 and Table 2 and not counted as one of our 177 studies as none of their results contributed to our analyses.

### 2.2. Data Management

The raw genomic, transcriptomic and proteomic data tables from 177 different studies were transformed into standardized tables for each disease on every omics layer. Different conversions were applied within this data management process, such as converting fold change to log_2_-fold change (log2FC), log_10_-*p*-value to *p*-value, the removal of entries with a missing gene name or separating rows that contained several gene names (proteomic data). Differences in multiple testing corrections were accepted, such as differences in the exact calculation of the fold change (log2FC, G-fold change). Only those genes with a false discovery ratio (FDR) ≤ 0.05 were selected after applying those conversions where necessary. Further, all genes that appeared as differentially expressed in only one experiment on the transcriptomics or proteomics level were discarded, to further reduce the number of genes that appeared randomly. Finally, all remaining genes from the genomic, transcriptomic and proteomic data sources intersected with the latest list of protein-coding gene symbols (04.08.20) from the HUGO Gene Nomenclature Committee to exclude non-standard gene names.

### 2.3. Data Analysis

We analyzed the gathered data in three different ways (see also Figure 2).

#### 2.3.1. Intersection

By intersecting the three analyzed omics layers per disease and the four diseases per omics layer, it is possible to test if the number of shared genes between some omics layers or diseases are significantly increased. We used a hypergeometric test to test the overlapping sets, with the total amount of 19,324 gene symbols of protein-coding genes (HUGO Gene Nomenclature Committee 04.08.20) [241] as the total population. Intersections were performed and visualized using the R (version 4.0.2) package venn (version 1.9) [242].

#### 2.3.2. Common Regulation between NDDs on a Transcriptomic Level

It was tested for the intersection of all NDDs on the transcriptomic level, if the direction of regulation for different NDDs was equal. The mean direction of regulation was computed as follows. Equation (1): Calculation of mean regulation of direction

(1) $$MeanRegDir\left(gene\right)=\frac{1}{n}\overset{n}{\underset{i=1}{\sum }}\left(sig\left(gen{e_{foldChange}}_{i}\right)\right)$$

with

n=number of appearences for gene with FDR ≤0.05

$$sig\left(x\right)=\;\{\begin{array}{c} 1,\;if\;x>0\\ -1,\;if\;x<0 \end{array}$$

In order to test for a correlation between the transcriptomic regulation of the four analyzed NDDs, a correlation test was performed using R’s cor.test() function. Additionally, the information about the mean direction of regulation (see. Equation (1)) of these genes was used to cluster the four analyzed NDDs based on the 139 genes appearing in the intersection of all transcriptomic data. Hierarchical clustering and creating a heatmap showing the results were performed using the R package pheatmap (version 1.0.12) [243].

#### 2.3.3. GO-Term- and Pathway Analyses

Independent of the test results, these sets of overlapping genes were also used for Kyoto Encyclopedia for Genes and Genomes (KEGG)-pathway analyses [244] and GO-Term [245] analyses. We used the R API WebGestaltR 0.4.4 of the online tool WebGestalt 2020 [246] to perform overrepresentation analyses (ORA) for all possible intersections per disease and per omics layer. For performing the ORA, the command WebGestaltR was used with the options:

- enrichDatabase = c(“pathway_KEGG”, “geneontology_Biological_Process”, “geneontology_Cellular_Component”, “geneontology_Molecular_Function”)
- interestGeneType = “genesymbol”
- referenceSet = “genome”
- topThr = 10000
- reportNum = 10000

The organism was set to “hsapiens” by default.

As the number of significantly overrepresented biological processes was very high in the ORA of the transcriptomic overlap for AD, PD, ALS and HD, the affinity propagation of the R package apcluster [247] was used. This method is already built in the WebGestalt tool and utilizes the affinity propagation method [248] to reduce the set of all biological processes to highly representative ones.

## 3. Results

### 3.1. Intersection

**Figure 3:**
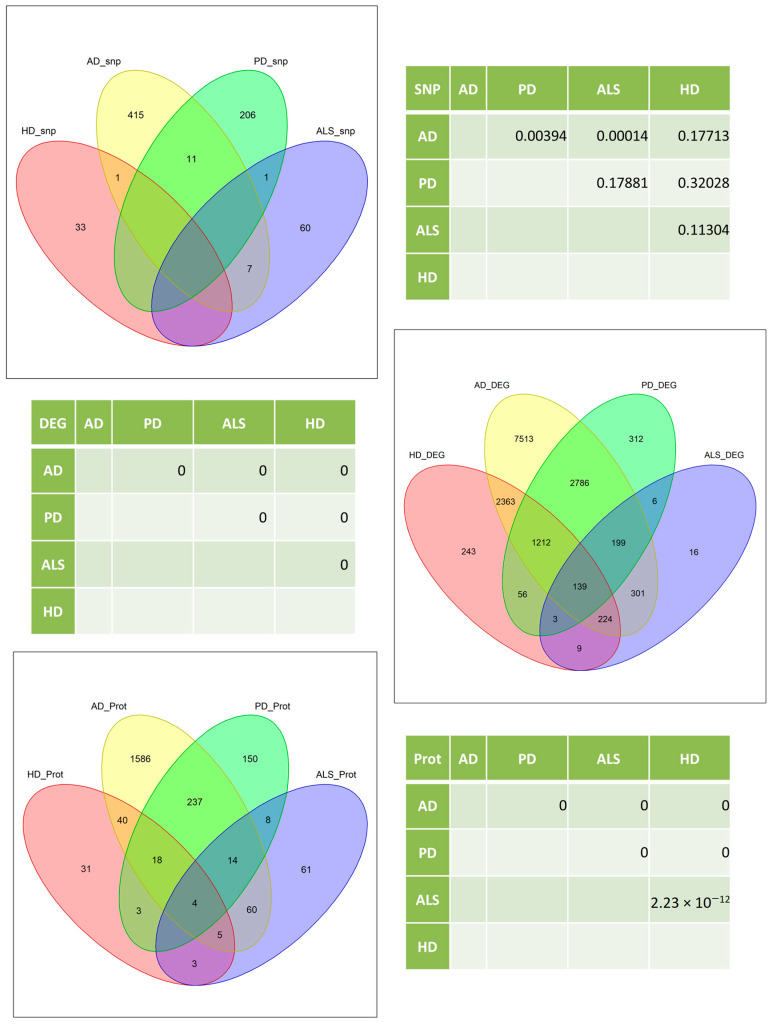
Venn diagrams and hypergeometric test results for the overlap between significant single nucleotide polymorphism (SNP)-trait associations (genomic level) and significantly differentially expressed genes on the transcriptomic (middle) and proteomic (bottom) levels for AD, PD, HD and ALS. All tested intersections show highly significant enriched numbers of overlapping genes for the transcriptomic and proteomic data. The genomic data show significantly enriched numbers of overlapping genes for the AD-PD and AD-ALS intersections.

To quantify if the number of overlapping genes between AD, PD, HD and ALS was high for one omics layer, a hypergeometric test was performed. The number of genes found in the GWAS Catalog was highest for AD, with 434 single nucleotide polymorphisms (SNPs). For PD, 218 SNPs were found; 68 were found for ALS and 34 SNPs for HD. The number of overlapping SNPs between each pair of diseases ranged from zero to eleven and was significantly high for the pairwise overlaps between AD and PD as well as AD and ALS in a hypergeometric test (see Figure 3). For the transcriptomic data, AD again showed the highest number of genes, with a total of 14,737 genes that were differentially expressed in at least two experiments. PD showed 4713 differentially expressed genes, ALS showed 897 and HD showed 4249. All pairwise comparisons of diseases on the transcriptomic level showed a highly significant enrichment in the number of overlapping genes. For AD, 1964 gene names could be related to differentially expressed proteins. We found 434 gene names for PD, 155 for ALS and 104 for HD. All pairwise overlaps between the four diseases were enriched for the proteome data with high significance.

### 3.2. Common Regulation of NDDs on the Transcriptomic Level

**Figure 4:**
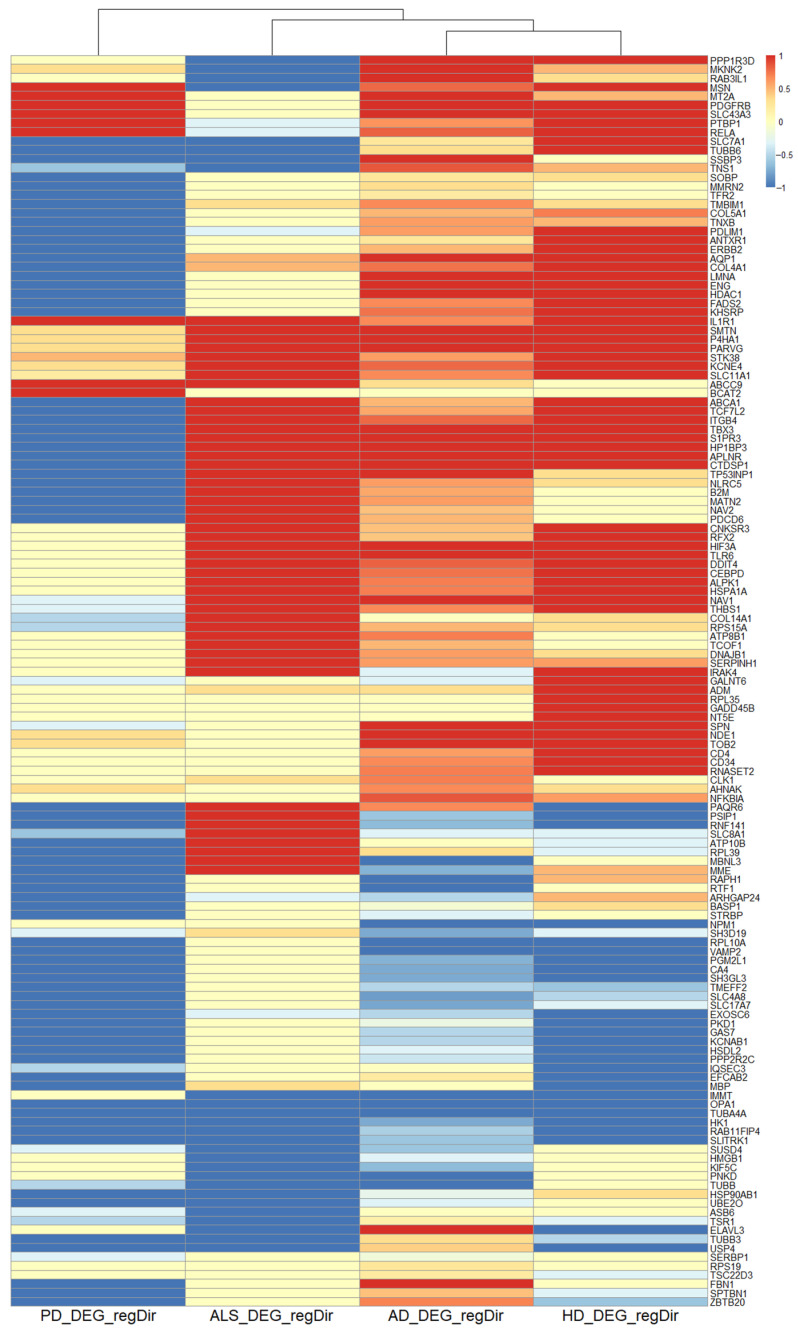
Heatmap with hierarchical clustering results of the mean regulation of all 139 genes that were maintained in all four NDD transcriptomic data. The clustering led to an inner cluster containing HD and AD transcriptomic data. This cluster was next clustered to the ALS transcriptomic data and finally these three NDD were clustered to PD. Colors represent the mean direction of regulation (see. Equation (1)).

The intersection of all four diseases’ transcriptomic data contained 139 genes. The hierarchical clustering result of the four NDDs based on these genes can be found in Figure 4 and shows an inner cluster formed of HD and AD consecutively extended by ALS and then PD.

The results of the correlation analysis are shown in Table 3. The most significant relation in terms of the mean direction of regulation existed between AD and HD with a *p*-value < 2.2 × 10^−16^. The correlation value of 0.657 showed a strong relation. All other pairwise comparisons showed a significant relation with correlation values > 0.3, except for the non-significant comparison between PD and ALS (correlation < 0.047).

### 3.3. GO-Term- and Pathway-Analyses

**Figure 5:**
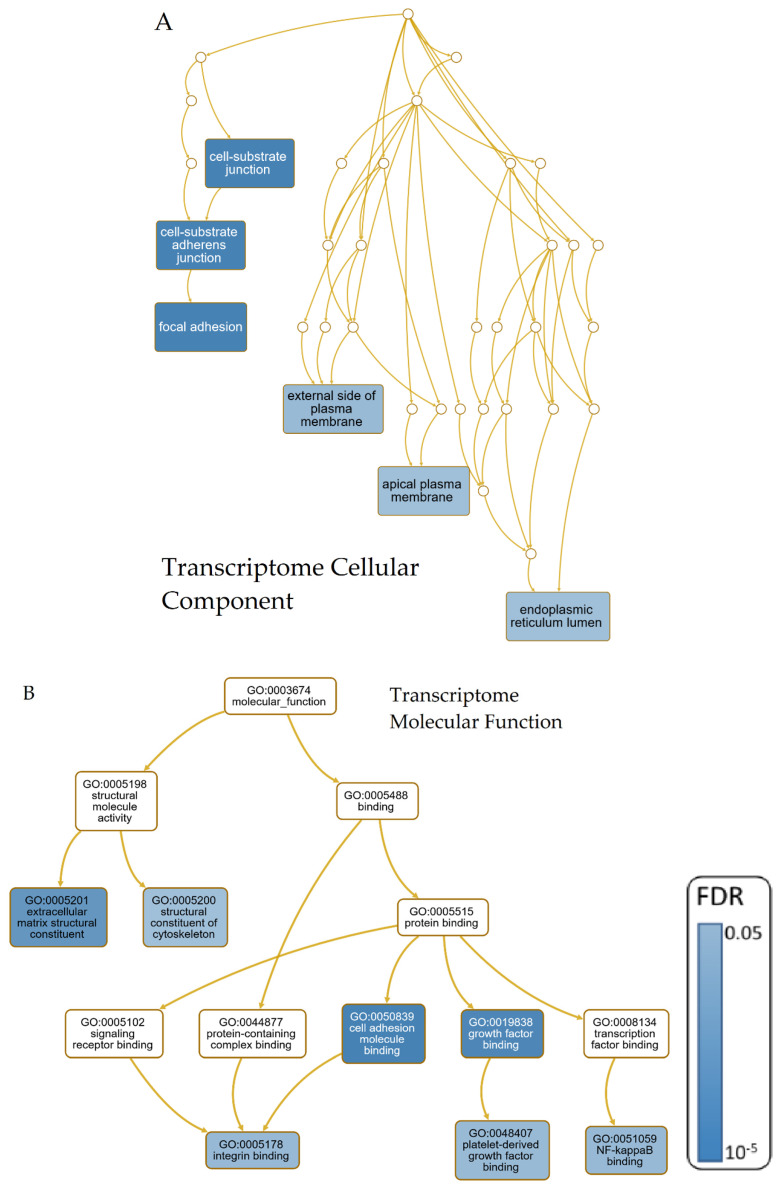

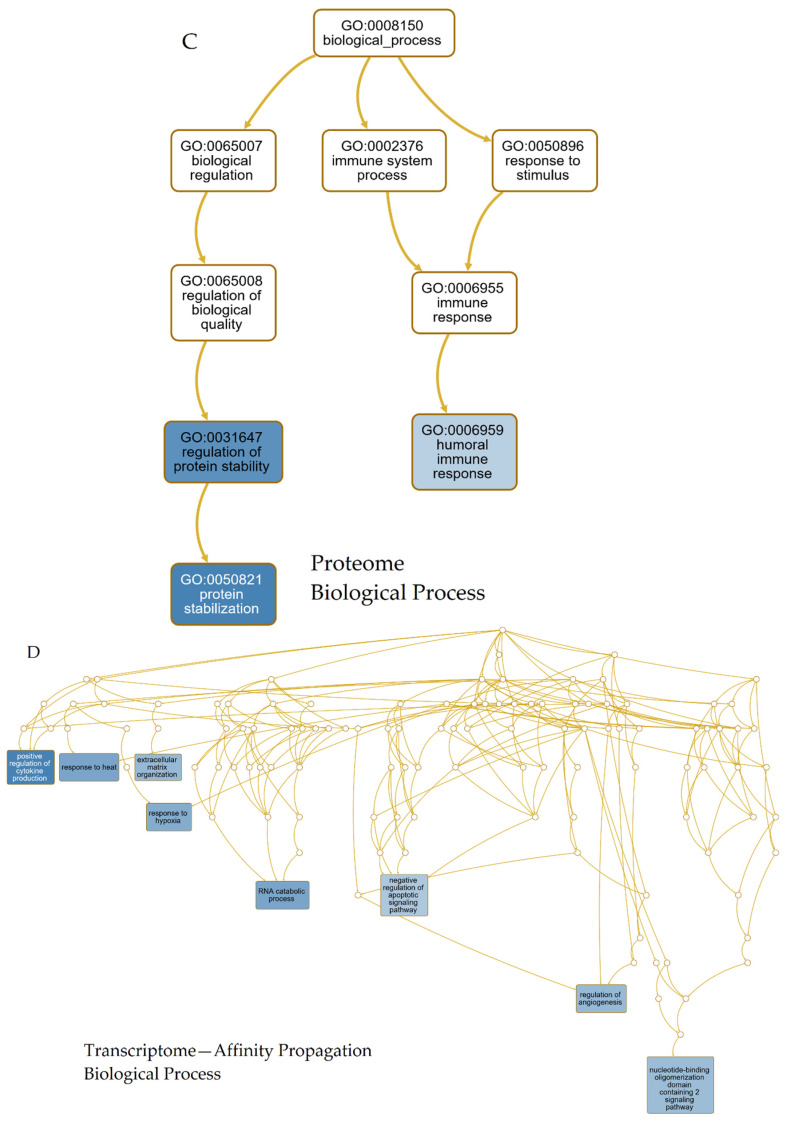
Directed acyclic graph showing the significant results (false discovery ratio (FDR) ≤ 0.05) of the GO-Term ORA of the Cellular Component and Molecular Function of the transcriptomic overlap of all four NDDs (**A**,**B**), the Biological Process terms of the proteomic data (**C**) and the Biological Process terms of the transcriptomic data after affinity propagation (**D**). Blue shading indicates the value of the FDR. For better readability, all GO-Terms leading to the significant ones were hidden in (**A**,**D**).

As the number of possible combinations of omics layers and diseases was very high, this study concentrated on describing the GO-Term and KEGG pathway analyses of the genes appearing in the intersection of all diseases per omics layer. GO-Terms and the contributing genes were analyzed in accordance with the conceptual model of candidate pathways contributing to neurodegeneration [15]. Results of the intersection of all four analyzed NDDs were also visualized as directed acyclic graphs that show the GO-Term hierarchy leading to the significant terms (Figure 5).

#### 3.3.1. Transcriptomic Intersection of AD, PD, ALS and HD

**Figure 6:**
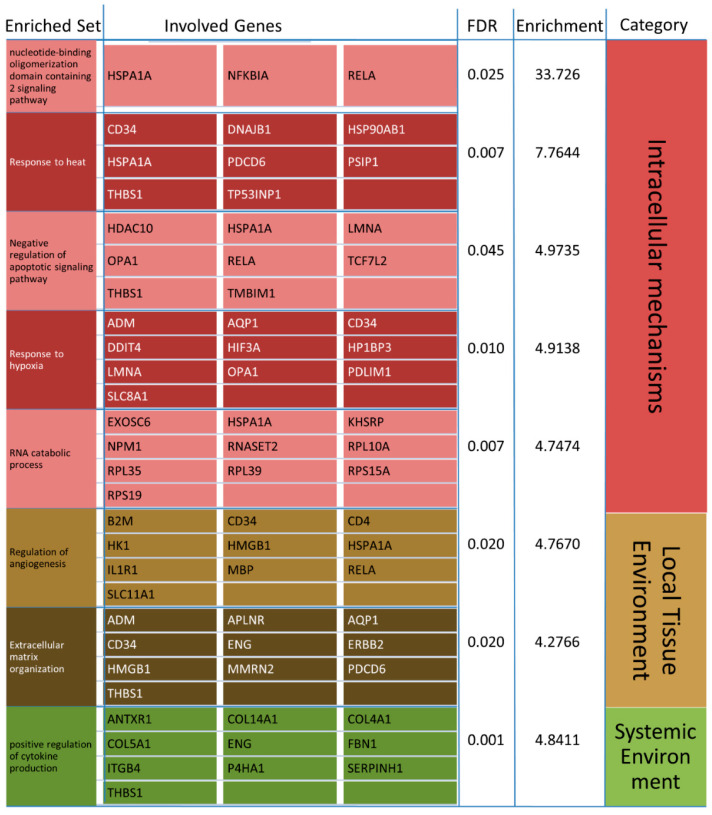
Significant results (FDR < 0.05) of the biological process (BP) GO-Term analysis for the transcriptomic overlap in the four analyzed NDDs after performing affinity propagation. The names of the enriched sets are shown in the left column, followed by the contributing genes on their right. For each significant set, the FDR and enrichment is given. The enriched BP sets are categorized in the groups Intracellular Mechanisms, Local Tissue Environment and Systemic Environment based on Ramanan’s conceptual model of candidate pathways contributing to neurodegeneration [15], which is also depicted in Figure 1.

The 28 overrepresented Biological Process (BP)-Terms for the intersection of all four diseases’ transcriptomic data were reduced to the eight most representative ones using affinity propagation (Figure 5D). All results of the GO-Term and KEGG pathway analyses performed are also given in the Supplementary File S5. The resulting enriched sets can mainly be related to cellular response to heat and stress (in our case hypoxia), but also the NOD2 signaling pathway, the negative regulation of apoptosis, a positive regulation of angiogenesis and cytokines, RNA catabolic processes and extracellular matrix organization (Figure 6). All of the six detected Cellular Component (CC)-Terms are related to focal adhesion, plasma membrane, and endoplasmic reticulum (ER) lumen (Figure 5A). The seven overrepresented Molecular Function (MF)-Terms can be categorized into structural molecule activity (structural constituent of cytoskeleton, extracellular matrix structural constituent) and protein binding (platelet-derived growth factor binding, growth factor binding, integrin binding, cell adhesion molecule binding, NF-κβ binding), as shown in Figure 5B. The KEGG pathway analysis showed prostate cancer as the only significant result (FDR = 0.025).

#### 3.3.2. Proteomic Intersection of AD, PD, ALS and HD

For the intersection of proteomic data for all four analyzed NDDs, three significantly overrepresented GO-Terms were found for BP (Figure 5C). Two of them are related to maintenance of protein stability and the third term to the immune system (humoral immune response). No significant results were discovered through MF, CC or the KEGG pathway analysis.

## 4. Discussion

### 4.1. Intersections

The number of shared genes with significant SNP-trait associations between the four NDDs on the genomic level was significantly enriched only for the AD-PD and AD-ALS comparisons. Interestingly, there was no single overlapping gene in the aforementioned study from 2018 on the association of genomic data between AD and PD and zero to two between AD and ALS, depending on the exact method [66]. For the proteomic and transcriptomic data, all numbers of pairwise overlapping genes were significantly enriched. However, the total number of overlapping genes between all four NDDs on the proteomic layer was rather low, with four genes, and did not exist at all on the genomic level. On the other hand, the transcriptomic data showed an overlap of 139 genes and thus allowed for a more distinctive analysis of GO-Terms, pathways and the concordance of regulation between the four analyzed NDDs. Nevertheless, the significance of all pairwise comparisons on the transcriptomic and proteomic levels confirms that there is a significant relation between all four analyzed NDDs—at least on the transcriptomic and proteomic level. The following evaluation of the GO-Term and KEGG pathway analysis was intended to reveal the nature of this relationship.

### 4.2. GO-Term and Pathway Analyses

#### 4.2.1. KEGG Pathway Analysis

As the result of the pathway analysis (prostate cancer) seemed unexpected as a common factor of neurodegeneration, we further researched the gene set leading to this KEGG pathway. Of the six genes in the prostate cancer pathway we found several genes that were both connected to cancer and neurodegeneration in general, including *NFKBIA, RELA* and *PDGFRB*. NF-κβ and RelA form a dimer with a transactivating domain that binds to specific DNA sequences as transcription factor controlling genes that are involved in immune and inflammatory responses and control of cell proliferation and apoptosis [249]. Misregulation of NF-κβ can lead to cancer [250], neurodegenerative [251], autoimmune and other inflammatory diseases [252].

Platelet-derived growth factor receptor β (PDGFRB) is a cell-surface receptor that plays an essential role in the regulation of cell proliferation, survival, differentiation, chemotaxis and migration, as well as in blood vessel development, where it can lead to uncontrolled blood vessel formation and cancer due to mutational activation or upregulation [253].

Consequently, at least half of the genes contributing to the prostate cancer gene set in our analysis are also associated with the formation of NDDs. Interestingly, a study in 2014 already described the existence of a significant overlap of genes described for either some types of cancer, such as prostate cancer, or NDDs such as AD and PD, based on their direction of regulation [254]. Nonetheless, the KEGG pathway analysis did not provide further insight into common factors of neurodegeneration.

The overrepresented GO-Terms for biological processes in the genes appearing in the intersection of all four NDDs on the transcriptomic level showed highly concordant results with a meta-study of AD, PD and ALS from 2019 [255] that analyzed the raw data of 259 individuals. That study found biological processes associated with heat shock proteins, cellular responses to heat, stress response and, additionally, GABA synthesis and protein folding, which were overrepresented in the four used datasets. They stated the importance of heat shock proteins (HSPs) as a general target of NDDs [256], and the importance of HSP-associated pathways in HD [257]. We, too, found cellular responses to heat and stress (in our case hypoxia), but also the NOD2 signaling pathway, the negative regulation of apoptosis, a positive regulation of angiogenesis and cytokines, RNA catabolic processes and extracellular matrix organization. However, no significant overrepresentation was found for GABA synthesis in our analysis of the intersection of the AD, PD, HD and ALS transcriptomic data. Protein stabilization as well as immune response was overrepresented in our comparison of the proteomic overlap of AD, PD, ALS and HD.

According to the literature, the GO-Term analysis revealed biological processes (reduced by affinity propagation) that are highly relevant to NDDs.

#### 4.2.2. Response to Heat

The cell’s response to heat is managed by heat shock proteins (HSPs), most of which are, despite of their names, expressed at average growth temperatures (37 °C). They belong to the cellular protein quality control and act as molecular chaperones to guide proteins from production to degradation. During aging, reduced amounts of HSPs and the increasing number of proteins requiring additional chaperoning can lead to an overstrained quality control system and ultimately to protein aggregation initiation [256].

#### 4.2.3. RNA Catabolic Process

Dysfunctional RNA catabolic processes have already been described for ALS and the nuclear RNA-binding protein TDP-43, which is integrally involved in RNA processing pathways, controlling the life cycle of RNAs from synthesis to degradation. In ALS, a cytoplasmic mislocalization and accumulation of TDP43 leads to TDP43 aggregates, misregulation of RNA processing and subsequent neuronal dysfunction [258,259]. Also, in AD aberrant phosphorylation, ubiquitination, cleavage and/or the nuclear depletion of TDP-43 in neurons and glial cells has been reported [260]. Our data suggest that the TDP-43 proteinopathy or another mechanism that leads to dysfunctional RNA catabolic processes plays a role in all four analyzed NDDs.

#### 4.2.4. Positive Regulation of Cytokine Production and Angiogenesis

The positive regulation of cytokine production and the regulation of angiogenesis are tightly connected because angiogenesis, the formation of new blood vessels from preexisting vessels, is partly induced by cytokines, as described for AD [261]. Amyloid-β plaques and neurofibrillary tangles induce activated microglia and elevated levels of pro-inflammatory cytokines [262]. Some of these cytokines, such as tumor necrosis factor-alpha (TNFα), interleukin (IL)-1β and transforming growth factor-β (TGF β) induce partly impaired angiogenesis, which builds up functional but also malfunctional vessels [263]. Due to decreased vascularity in the aging brain, hypoxia also stimulates the angiogenic process and endothelial activation. Activated endothelial cells elaborate several proteases, inflammatory factors and other products with biologic activity that may promote neuronal death [264,265]. Due to our findings, it can be assumed that protein aggregation in any of the four analyzed NDDs leads to neuroinflammation, which is accompanied by upregulated cytokines and impaired subsequent angiogenesis. As pro- and antiangiogenic factors regulate angiogenesis, both, cytokines and cytokine blockades could serve as potential pharmaceutical targets modulating angiogenesis in chronic inflammation [263,266].

#### 4.2.5. Response to Hypoxia

Hypoxia is a well-described multifaceted cause of NDDs. As mentioned above, aging and brain injuries like small infarcts lead to lower oxygen levels in the brain. During hypoxic events, high levels of free oxygen and nitrogen radicals are produced through mitochondrial complex III, which cannot be compensated for due to lower levels of antioxidants in aging and diseased brains, thus leading to the oxidative damage of vital cellular components [267]. Also, the impaired cellular homeostasis of metals like Ca^2+^ can be triggered by hypoxic conditions, resulting in changes in excitation and the inhibition of neuronal and glial cells. Synaptic transmission in the central nervous system (CNS) is susceptible to hypoxia, as it requires 30–50% of cerebral oxygen. Already very early during age-related hypoxia, a decrease in synaptic efficacy occurs [268].

#### 4.2.6. Extracellular Matrix Organization

Extracellular matrix (ECM) molecules in the central nervous system form highly organized structures around cell somata, axon initial segments, and synapses. They play prominent roles in early development by guiding cell migration, neurite outgrowth and synaptogenesis, and by regulating synaptic plasticity and stability, cognitive flexibility and axonal regeneration in adults. Upregulation of ECM molecules—in particular through reactive astrocytes, after brain injuries and during aging, neuroinflammation and neurodegeneration—results in the formation of a growth-impermissive environment and impaired synaptic plasticity. Thus, targeting the expression of specific ECM molecules, associated glycans and degrading enzymes may lead to the development of new therapeutic strategies promoting regeneration and synaptic plasticity [269].

#### 4.2.7. Nucleotide-Binding Oligomerization Domain Containing 2 Signaling Pathway

The nucleotide-binding oligomerization domain containing 2 of the NOD2 signaling pathway is part of the immune response by recognizing bacteria with a muramyl dipeptide (MDP) moiety and thus activating the transcription factor NF-κβ, which regulates the transcription of a large number of genes, especially those involved in the immune and inflammatory response, control of apoptosis and cell proliferation. Misregulation of NF-κβ can lead to cancer, but also to NDDs and other inflammatory diseases. Studies have showed that the E3 ubiquitin ligase parkin targets NOD2 for ubiquitinylation and subsequent degradation in order to regulate astrocyte endoplasmic reticulum stress and inflammation. Mutations in the Parkin gene, which are one reason for familial PD, lead to an overrepresentation of NOD2 [270]. Also, bacterial and viral infections are a known cause of AD. In our study, the NOD2 signaling pathway was significantly overrepresented for the intersection of all four NDDs’ transcriptomic data, leading to the hypothesis that bacterial or viral immune response might be a crucial factor for PD, HD and ALS as well.

#### 4.2.8. Negative Regulation of Apoptotic Signaling Pathway

Seven of the eight found BP after affinity propagation for all four NDDs’ transcriptomic data, seemed concordant with the literature. Also, the CC, which are mainly about focal adhesion but also about the cell-substrate adherens junction, cell-substrate junction, external side of plasma membrane, endoplasmic reticulum lumen and apical plasma membrane, fit very well to the BP. The significantly overrepresented MF, which are extracellular matrix structural constituent, cell adhesion molecule binding, structural constituent of cytoskeleton, integrin binding, platelet-derived growth factor binding, growth factor binding and NF-κβ binding are in accordance to the BP. Only the negative regulation of the (extrinsic) apoptotic signaling pathway seems surprising, as enhanced intrinsic or extrinsic apoptosis is typical of NDDs, leading to the severe loss of neurons that characterizes these diseases. A lower rate of apoptosis is a typical hallmark of cancer where even damaged cells are not abolished. However, ORA is based on a set of genes not taking the direction of their regulation into account. This direction of regulation was to some extent heterogenous between the four analyzed NDDs (see Figure 4) and, for each of the four NDDs, some of the genes contributing to the gene set was downregulated for the negative regulation of apoptotic signaling pathways. Consequently, the direction of regulation stated in the BP terms is not directly linked to the true direction of regulation in the NDDs.

#### 4.2.9. Protein Stabilization and Regulation of Protein Stability

Regulation of protein stability is the top-level term for the maintenance of unfolded protein, protein destabilization and protein stabilization. Unfolded proteins are a common characteristic of neurodegenerative diseases, as the accumulation of misfolded proteins causes stress response mechanisms in the endoplasmic reticulum (ER) [271]. Chronical ER stress caused by protein accumulation can lead to the initiation of apoptosis and, consequently, neurotoxicity [272]. Protein stabilization of TDP-43 has been described as one of the underlying factors of neuronal TDP-43-dependent toxicity in ALS and frontotemporal dementia [273]. It can be concluded that alterations in the general regulation of protein stability are described in both the general formation of neurodegeneration and the specific mechanisms for single NDDs, such as ALS. Additionally, the ER was also present as a cellular component that was significantly overrepresented in the transcriptomic gene set, which further enhances the idea of ER stress being involved as a common factor of neurodegeneration.

#### 4.2.10. Humoral Immune Response

The central nervous system (CNS) has always been considered to be the sole domain of the innate immune response rendered by the microglia. However, immune cells are increasingly recognized as being able to access to the CNS in both health and disease [274]. Lymphocytes can enter the CNS through the blood–brain barrier (BBB), the blood-meningeal barrier and the blood-cerebrospinal fluid (CSF) barrier [275,276]. Under healthy steady-state conditions, B cells are present in very low numbers in the CNS parenchyma and CSF [277], but in cases of CNS inflammation like multiple sclerosis, B cell numbers can increase by at least several orders of magnitude in the CNS parenchyma and perivascular spaces, and by severalfold in the CSF [278,279]. B cell-depleting therapy in patients with multiple sclerosis with rituximab and ocrelizumab has reduced inflammation significantly [274]. Understanding how the adaptive immune system participates in the pathogenesis of NDDs might deliver new possibilities for their treatment.

For PD, there is evidence that humoral immune response has been involved in course of the disease. Although B cells have not been detected in the brains of patients with PD [280], deposits of immunoglobulin G (IgG) have been found on the dopaminergic neurons in these patients, and Lewy bodies themselves are coated with IgG [281], which suggests that dopaminergic neurons might be targeted by these immunoglobulins.

In AD, the adaptive immune system could, apart from a possible involvement of plaque removal, be responsible for the immune response of infections with herpesviruses and other pathogens, which are acknowledged to be a possible source of AD. Our data suggest that the humoral immune response could be a target for further investigations in HD and ALS as well.

Interestingly, the GO-Term analysis showed an overall differing result between the proteomic and transcriptomic data. The BP terms for protein folding and the immune system are also highly consistent with the knowledge about the origin of neurodegeneration, as well as the findings that emerged from the transcriptomic analyses, but it is noteworthy that the analysis of the proteome contains characteristic findings that extend the transcriptomic insights.

#### 4.2.11. Common Regulation on the Transcriptomic Level

The hierarchical clustering of these 139 genes formed an inner cluster consisting of AD and HD (Figure 4). The correlation analysis showed a highly significant relation between these 139 genes for AD and HD with a high correlation value (*p*-value < 2.2 × 10^−16^ correlation: 0.66). This result shows that the set representing the least common denominator of the four NDDs is regulated in a very similar manner in HD and AD. Although this does not necessarily say anything about the significance of these genes in the course of the development of individual diseases, the high degree of concordance between the biological processes we have just described (Figure 6) for the 139 genes appearing to be significantly differentially regulated in all four analyzed NDDs and the actual development of neurodegeneration is striking.

## 5. Conclusions

GWA studies significantly contributed to understanding NDDs over the last 15 years, with several-hundred disease-associated risk loci. However, no targeted therapies have emerged from these GWA studies for most NDDs [282]. As NDDs are causing profound transcriptomic changes in the aging brain, it is crucial to take transcriptomic data analysis into account when analyzing NDDs [283]. Further translation from transcriptomic changes to proteomic occurs only indirectly and shows only a limited correlation between mRNA and protein expression [284]. Consequently, even the combination of genetic and transcriptomic data is not adequate to give a complete picture of the changes taking place due to NDDs. Each additional level of information can contribute to a better understanding of the complex interrelationships of these interacting omics layers.

To address the challenges of such complicated diseases, the whole field of biomedicine is changing towards creating and facilitating a variety of databases and analysis pipelines for separate omics layers and multi-omics integration [285]. Many of these pipelines are mainly data-driven and enable clustering and supervised machine learning techniques to find essential patterns of features contributing to the identification of, for example, proteins that are associated with NDDs [286], or to reveal cross-talk patterns in multi-omics data [287].

According to the necessity of approaching complex diseases with the use of multiple omics-layers, data-driven methods and large amounts of data, we combined the data of three omics layers from databases and literature mining of more than 1 million subjects and 177 studies to show the shared genes between the four analyzed NDDs and extract the pathways and processes in which they are overrepresented.

To classify the gained information in this study, it is crucial to keep in mind that the transcriptomic and proteomic data were gathered from various tissues, partly different severities of diseases and using different methods. Ninety percent of the non-control patients in the proteomic data and 84% in the transcriptomic data were classified with a moderate or severe disease state. Of the experiments, 63% in the transcriptomic data and 67% in the proteomic data were conducted with brain material, while the remaining were conducted with blood, spinal cord, cerebral spinal fluid or induced pluripotent stem-cells. A detailed table can be found in the Supplementary materials (S4). Consequently, the signals in this meta-study represent stable signals found mainly across brain tissues emerging in the late stage of NDDs, rather than subtle effects that might only be present in just one specific brain region or in earlier disease states. In accordance with the law of large numbers, meta-studies like ours are particularly well-suited to finding the results that represent real effects in partly noisy data. Such real effects occur repeatedly and were therefore able to exceed our own defined threshold of occurrences in two different experiments, thus contributing to further analyses. Even in the ORA, subtle effects of noisy data (e.g., from early stage disease studies) that were still present would probably be canceled out by the number of other strong signals and the fact that the majority of the used data are based on late stage NDDs.

The highly significant overrepresentation of genes in the intersection of proteomic and transcriptomic data in all investigated NDDs shows the importance of simultaneously analyzing multiple omics layers. Additionally, this analysis showed the relevance of questioning old results using updated databases. While no single overlapping gene was found in 2018 between the genomic data of AD and ALS, and only two were found between AD and PD [66], the same analysis led to significant overlaps between AD and PD and AD and ALS. The common factor relating to neurodegeneration from a 2019 study [255] that used the data of 259 samples could partly be confirmed. We also found biological processes for response to heat and stress (hypoxia) as well as protein folding to be significantly overrepresented. However, we could not reproduce the finding of an overrepresentation of the GABA synthesis pathway or any related terms. Additionally, the NOD2 signaling pathway, the negative regulation of apoptosis, positive regulation of cytokines and angiogenesis, RNA catabolic processes, extracellular matrix organization and humoral immune response emerged from our analysis. All of these results emerging from the GO-Term analysis of the transcriptomic and proteomic data seem highly plausible as common factors of neurodegeneration, and shed light on processes like humoral immune response that have previously been described only for certain diseases.

Accordingly, this meta-study reveals highly significant processes common to all analyzed NDDs and might therefor contribute to the development of pharmaceutical measures against neurodegeneration in general. Regarding future research on this topic, it might be helpful to expand the repertoire of omics layers by epigenomics and concentrate further on the differences between these separate NDDs according to the regulation of these common genes. Additional analysis of the overlap between different omics layers at the level of individual diseases, as well as differences in the intersection of AD, PD and ALS in contrast with the autosomal dominant disorder HD could also provide new insight in light of knowledge of the processes common to all NDDs.

## Figures and Tables

**Table 1:** Overview of the number of cases and controls and the total number of studies per disease throughout all analyzed transcriptome studies. In total, data of 2181. Samples were gathered from 39 studies analyzing transcriptomic data. * One study conducted experiments for AD and HD. Thus, in total proteomic data of 22 studies was used.

Transcriptome	Case	Control	Sum of Samples	Studies
AD	187	194	381	11
PD	252	215	467	11
HD	73	99	173	10
ALS	470	691	1161	8
∑	982	1199	2181	40 (39 *)

**Table 2:** Overview of the number of cases, controls and the total number of studies per disease throughout all analyzed proteome studies. In total, data of 1969 samples were gathered from 22 studies analyzing proteomic data. * Two studies conducted experiments for AD and PD. Thus, in total proteomic data of 22 studies was used.

Proteome	Case	Control	Sum of samples	Studies
AD	853	444	1297	9
PD	146	167	313	7
HD	39	29	68	5
ALS	162	129	291	3
∑	1200	769	1969	24 (22 *)

**Table 3:** Results of the one stratum analysis of the linear regression model calculated with the cor.test function in R. The *p*-value and correlation values are given for all pairwise comparisons of the analyzed NDDs. The analyzed values are the mean direction of regulation of all transcriptomic data kept after intersecting the four NDDs. *p*-Values < 0.05 are shown in red.

NDD	AD-PD	AD-ALS	AD-HD	PD-ALS	PD-HD	ALS-HD
*p*-value	0.0001185	0.000131	<2.2 × 10^−16^	0.5887	2.422 × 10^−6^	0.0002744
Correlation	0.320714	0.3187755	0.6566416	0.04625543	0.387635	0.3040112

## Supplementary Materials

The following are available online at https://www.mdpi.com/2073-4409/9/12/2642/s1, Table S1: Overview of studies used for gathering genomic data—GWAS Catalog study information, Table S2: Overview of studies used for gathering transcriptomic data—Transcriptome study information, Table S3: Overview of studies used for gathering proteomic data— Proteome study information, File S4: NDD_Disease State and Tissues, File S5: GO-Term and Pathway Analyses.
