# Supplementary material for: Common Factors in Neurodegeneration: A Meta-Study Revealing Shared Patterns on a Multi-Omics Scale
Source: Cells. 2020 Dec 8;9(12):2642. doi: 10.3390/cells9122642 (PMC7764447; doi:10.3390/cells9122642)
Supplement: Supplementary file 1 [file cells-09-02642-s001.zip › Supplementary/Supplementary Tables.pdf]

cells

MDPI

Supplementary Tables

Table S1. Overview of studies used for gathering transcriptomic data—GWAS Catalog study information. AD = Alzheimer's disease, PD = Parkinson's disease, (s)ALS =(sporadic) Amyotrophic Lateral Sclerosis, HD = Huntington's disease, ctrl = control, CSF = cerebrospinal fluid, BA = Brodman Area.

Study	Participants	Comparison	Paper	Disease
(Nazarian, Yashin and Kulminski, 2019)	952 European ancestry male cases, 1,789 European ancestry female cases, 6,337 European ancestry male controls, 8,402 European ancestry female controls	NA	Genome-wide analysis of genetic predisposition to Alzheimer's disease and related sex disparities.	Alzheimer's disease
(Nazarian *et al.*, 2019)	796 European ancestry cases, 4,010 European ancestry controls	NA	Genetic heterogeneity of Alzheimer's disease in subjects with and without hypertension.	Alzheimer's disease
(Beecham *et al.*, 2009)	492 European ancestry cases, 496 European ancestry controls	238 European ancestry cases, 220 European ancestry controls	Genome-wide association study implicates a chromosome 12 risk locus for late-onset Alzheimer disease.	Alzheimer's disease
(Carrasquillo *et al.*, 2009)	844 European ancestry cases, 1,255 European ancestry controls	1,547 European ancestry cases, 1,209 European ancestry controls	Genetic variation in *PCDH11X* is associated with susceptibility to late-onset Alzheimer's disease.	Alzheimer's disease
(Abraham *et al.*, 2008)	1,082 European ancestry cases, 1,239 European ancestry controls	1,400 controls	A genome-wide association study for late-onset Alzheimer's disease using DNA pooling.	Alzheimer's disease
(Webster *et al.*, 2007)	664 cases, 422 controls	NA	*SORL1* as an Alzheimer's disease predisposition gene?	Alzheimer's disease
(Coon *et al.*, 2007)	664 European ancestry cases, 422 European ancestry controls	NA	A high-density whole-genome association study reveals that *APOE* is the major susceptibility gene for sporadic late-onset Alzheimer's disease.	Alzheimer's disease
(Heinzen *et al.*, 2010)	331 European ancestry cases, 368	NA	Genome-wide scan of copy number variation	Alzheimer's disease

	European ancestry controls		in late-onset Alzheimer's disease.	
(Naj *et al.*, 2010)	931 cases, 1,104 controls	1,338 cases, 2,003 controls	Dementia revealed: novel chromosome 6 locus for late-onset Alzheimer disease provides genetic evidence for folate-pathway abnormalities.	Alzheimer's disease
(Logue *et al.*, 2011)	513 African American cases, 496 African American controls	NA	A comprehensive genetic association study of Alzheimer disease in African Americans.	Alzheimer's disease
(Hu *et al.*, 2011)	1,831 European ancestry cases, 1,764 European ancestry controls	751 cases, 751 controls	Meta-analysis for genome-wide association study identifies multiple variants at the BIN1 locus associated with late-onset Alzheimer's disease.	Alzheimer's disease
(Sherva *et al.*, 2014)	303 European ancestry cases		Genome-wide association study of the rate of cognitive decline in Alzheimer's disease.	Alzheimer's disease
(Lo *et al.*, 2019)	2,399 European ancestry cases, 4,160 European ancestry controls	NA	Identification of genetic heterogeneity of Alzheimer's disease across age.	Alzheimer's disease
(Zhu *et al.*, 2019)	54,162 European ancestry cases, 58,047 European ancestry individuals	NA	Shared genetic architecture between metabolic traits and Alzheimer's disease: a large-scale genome-wide cross-trait analysis.	Alzheimer's disease
(Feulner *et al.*, 2010)	491 European ancestry cases, 479 European ancestry controls	NA	Examination of the current top candidate genes for AD in a genome-wide association study.	Alzheimer's disease
(Kramer *et al.*, 2011)	185 European ancestry low NFT individuals, 114 European ancestry high NFT individuals	NA	Alzheimer disease pathology in cognitively healthy elderly: a genome-wide study.	Alzheimer's disease

(Meda *et al.*, 2012)	367 European ancestry individuals with mild cognitive impairment, 181 European ancestry individuals with mild early-stage LOAD, 209 European ancestry controls	NA	A large scale multivariate parallel ICA method reveals novel imaging-genetic relationships for Alzheimer's disease in the ADNI cohort.	Alzheimer's disease
(Kamboh, Barmada, *et al.*, 2012)	1,190 European ancestry cases, 1,032 cases	NA	Genome-wide association analysis of age-at-onset in Alzheimer's disease.	Alzheimer's disease
(Chung *et al.*, 2018)	190 European ancestry Alzheimer's disease dementia cases	NA	Genome-wide association study of Alzheimer's disease endophenotypes at prediagnosis stages.	Alzheimer's disease
(Nazarian *et al.*, 2019)	1,262 European ancestry cases, 9,608 European ancestry controls	NA	Genetic heterogeneity of Alzheimer's disease in subjects with and without hypertension.	Alzheimer's disease
(Herold *et al.*, 2016)	2,478 European ancestry cases, 979 ancestry controls both from the same 1,070 families	NA	Family-based association analyses of imputed genotypes reveal genome-wide significant association of Alzheimer's disease with OSBPL6, PTPRG, and PDCL3.	Alzheimer's disease
(Cummings *et al.*, 2012)	109 Amish cases, 689 Amish controls	NA	Genome-wide association and linkage study in the Amish detects a novel candidate late-onset Alzheimer disease gene.	Alzheimer's disease
(Tosto *et al.*, 2015)	2,451 Caribbean Hispanic cases, 2,063 Caribbean Hispanic controls	550 Caribbean Hispanic cases, 236 Caribbean Hispanic controls	F-box/LRR-repeat protein 7 is genetically associated with Alzheimer's disease.	Alzheimer's disease
(Pérez-Palma *et al.*, 2014)	2,540 European ancestry cases, 2,029 European ancestry controls	NA	Overrepresentation of glutamate signaling in Alzheimer's disease: network-based pathway enrichment using meta-analysis of	Alzheimer's disease

			genome-wide association studies.	
(Wang *et al.*, 2015)	983 cases	NA	Genetic Determinants of Survival in Patientswith Alzheimer's Disease.	Alzheimer's disease
(Yashin *et al.*, 2018)	12,169 European ancestry individuals, 2,078 Black individuals, 4,053 unknown ancestry individuals	NA	Hidden heterogeneity in Alzheimer's disease: Insights from genetic association studies and other analyses.	Alzheimer's disease
(Reiman *et al.*, 2007)	446 cases, 290 controls	415 cases, 260 controls	GAB2 alleles modify Alzheimer's risk in APOE epsilon4 carriers.	Alzheimer's disease
(Kim *et al.*, 2011)	96 European ancestry Alzheimer disease cases, 176 European ancestry individuals with mild cognitive impairment, 102 European ancestry controls	NA	Genome-wide association study of CSF biomarkers Abeta1-42, t-tau, and p-tau181p in the ADNI cohort.	Alzheimer's disease
(Kamboh, Demirci, *et al.*, 2012)	1,291 European ancestry cases, 938 European ancestry controls	509 European ancestry cases, 753 European ancestry controls, 2,218 cases, 2,583 controls	Genome-wide association study of Alzheimer's disease.	Alzheimer's disease
(Stein *et al.*, 2010)	173 European ancestry Alzheimer's disease cases, 361 European ancestry Mild Cognitive Impairment cases, 208 European ancestry controls	NA	Genome-wide analysis reveals novel genes influencing temporal lobe structure with relevance to neurodegeneration in Alzheimer's disease.	Alzheimer's disease
(Martinelli-Boneschi *et al.*, 2013)	92 European ancestry cases, 77 European ancestry controls	94 European ancestry cases, 74 European ancestry controls	Pharmacogenomics in Alzheimer's disease: a genome-wide association study of response to cholinesterase inhibitors.	Alzheimer's disease

(Bertram *et al.*, 2008)	941 European ancestry cases and 404 European ancestry controls from 410 families	1,767 European ancestry cases and 838 European ancestry controls from 875 families	Genome-wide association analysis reveals putative Alzheimer's disease susceptibility loci in addition to APOE.	Alzheimer's disease
(Lo *et al.*, 2019)	7,316 European ancestry cases, 7,579 European ancestry controls	NA	Identification of genetic heterogeneity of Alzheimer's disease across age.	Alzheimer's disease
(Ramanan *et al.*, 2014)	555 European ancestry individuals	NA	APOE and BCHE as modulators of cerebral amyloid deposition: a florbetapir PET genome-wide association study.	Alzheimer's disease
(Chung *et al.*, 2018)	190 European ancestry AD dementia cases	NA	Genome-wide association study of Alzheimer's disease endophenotypes at prediagnosis stages.	Alzheimer's disease
(Jonsson *et al.*, 2013)	3,550 European ancestry cases, 8,888 European ancestry controls	694 European ancestry cases, 4,375 European ancestry controls, 1,343 cases, 5,352 controls	Variant of TREM2 associated with the risk of Alzheimer's disease.	Alzheimer's disease
(Lee *et al.*, 2017)	242 European ancestry individuals	NA	Single-nucleotide polymorphisms are associated with cognitive decline at Alzheimer's disease conversion within mild cognitive impairment patients.	Alzheimer's disease
(Marioni *et al.*, 2018)	up to 42,034 British ancestry individuals with parental history of Alzheimer's disease, at least 272,244 British ancestry individuals with no parental history of Alzheimer's disease, 25,580 Alzheimer's	NA	GWAS on family history of Alzheimer's disease.	Alzheimer's disease

	disease cases, 48,466 controls			
(Traylor *et al.*, 2016)	17,008 European ancestry Alzheimer's disease cases, 3,651 European ancestry small vessel stroke cases, 95,811 European ancestry controls	NA	Shared genetic contribution to Ischaemic Stroke and Alzheimer's Disease.	Alzheimer's disease
(Furney *et al.*, 2011)	424 European ancestry mild cognitive impairment cases, 236 European ancestry Alzheimer's disease cases, 279 European ancestry controls	NA	Genome-wide association with MRI atrophy measures as a quantitative trait locus for Alzheimer's disease.	Alzheimer's disease
(Hirano *et al.*, 2015)	489 Japanese ancestry APOE-ε4 noncarrier cases, 6,463 Japanese ancestry APOE-ε4 noncarrier controls, 323 Japanese ancestry APOE-ε4 carrier cases, 1,484 Japanese ancestry APOE-ε4 carrier controls, 4 Japanese ancestry cases, 45 Japanese ancestry controls	528 Japanese ancestry APOE-ε4 noncarrier cases, 5,824 Japanese ancestry APOE-ε4 noncarrier controls, 480 Japanese ancestry APOE-ε4 carrier cases, 1,364 Japanese ancestry APOE-ε4 carrier controls, 3 Japanese ancestry cases, 24 Japanese ancestry controls	A genome-wide association study of late-onset Alzheimer's disease in a Japanese population.	Alzheimer's disease
(Wijsman *et al.*, 2011)	1,848 European ancestry affected individuals, 1,991 European ancestry unaffected individuals	231 Caribbean Hispanic cases, 187 Caribbean Hispanic controls, 386 European ancestry cases, 386 European	Genome-wide association of familial late-onset Alzheimer's disease replicates BIN1 and CLU and nominates CUGBP2 in interaction with APOE.	Alzheimer's disease

		ancestry controls		
(Kauwe *et al.*, 2014)	574 individuals	NA	Genome-wide association study of CSF levels of 59 alzheimer's disease candidate proteins: significant associations with proteins involved in amyloid processing and inflammation.	Alzheimer's disease
(Deming *et al.*, 2016)	123 Alzheimer's disease cases, 270 controls	NA	A potential endophenotype for Alzheimer's disease: cerebrospinal fluid clusterin.	Alzheimer's disease
(Mukherjee *et al.*, 2018)	Up to 1,107 European ancestry cases, 3,447 European ancestry controls	NA	Genetic data and cognitively defined late-onset Alzheimer's disease subgroups.	Alzheimer's disease
(Antúnez *et al.*, 2011)	319 European ancestry cases, 769 European ancestry controls, 2,690 cases, 2,237 controls	4,982 European ancestry cases, 7,961 European ancestry controls, 2,190 cases, 3,374 controls	The membrane-spanning 4-domains, subfamily A (MS4A) gene cluster contains a common variant associated with Alzheimer's disease.	Alzheimer's disease
(Lambert *et al.*, 2009)	2,032 European ancestry cases, 5,328 European ancestry controls	3,978 European ancestry cases, 3,297 European ancestry controls	Genome-wide association study identifies variants at CLU and CR1 associated with Alzheimer's disease.	Alzheimer's disease
(Li *et al.*, 2008)	753 European ancestry cases, 736 European ancestry controls	418 European ancestry cases, 249 European ancestry controls	Candidate single-nucleotide polymorphisms from a genomewide association study of Alzheimer disease.	Alzheimer's disease
(Miyashita *et al.*, 2013)	891 Japanese ancestry cases, 844 Japanese ancestry controls	1,224 East Asian ancestry cases, 2,114 East Asian ancestry controls, 11,840 European ancestry cases, 10,931 European ancestry controls	SORL1 is genetically associated with late-onset Alzheimer's disease in Japanese, Koreans and Caucasians.	Alzheimer's disease

(Cruchaga *et al.*, 2013)	591 European ancestry cases, 687 European ancestry controls		GWAS of cerebrospinal fluid tau levels identifies risk variants for Alzheimer's disease.	Alzheimer's disease
(Reitz *et al.*, 2013)	1,968 African American cases, 3,928 African American controls		Variants in the ATP-binding cassette transporter (ABCA7), apolipoprotein E ϵ4,and the risk of late-onset Alzheimer disease in African Americans.	Alzheimer's disease
(Hollingworth *et al.*, 2012)	1,039 European ancestry cases with psychosis, 5,659 European ancestry controls, 260 European, African American and Native American ancestry cases with psychosis from 264 families	NA	Genome-wide association study of Alzheimer's disease with psychotic symptoms.	Alzheimer's disease
(Seshadri *et al.*, 2010)	973 incident AD cases, 2,033 prevalent AD cases, 22,604 controls of European and Hispanic ancestry	6,505 European ancestry cases, 13,532 European ancestry controls	Genome-wide analysis of genetic loci associated with Alzheimer disease.	Alzheimer's disease
(Gusareva *et al.*, 2018)	788 male cases, 1,455 female cases, 2,362 male controls, 3,655 female controls	3,836 male cases, 6,244 female cases, 8,618 male controls, 11,624 female controls	Male-specific epistasis between WWC1 and TLN2 genes is associated with Alzheimer's disease.	Alzheimer's disease
(Nelson *et al.*, 2014)	1443 cases and 99 controls	NA	ABCC9 gene polymorphism is associated with hippocampal sclerosis of aging pathology.	Alzheimer's disease
(Jansen *et al.*, 2019)	24,087 European ancestry late-onset Alzheimer's disease cases, 47,793 European ancestry individuals with family history of Alzheimer's	NA	Genome-wide meta-analysis identifies new loci and functional pathways influencing Alzheimer's disease risk.	Alzheimer's disease

	disease, 383,378 European ancestry controls			
(Moreno-Grau *et al.*, 2019)	4,120 European ancestry cases, 3,289 European ancestry controls	NA	Genome-wide association analysis of dementia and its clinical endophenotypes reveal novel loci associated with Alzheimer's disease and three causality networks: The GR@ACE project.	Alzheimer's disease
(Schott *et al.*, 2016)	293 cases, 10,547 healthy controls	NA	Genetic risk factors for the posterior cortical atrophy variant of Alzheimer's disease.	Alzheimer's disease
(Ramirez *et al.*, 2014)	363 European ancestry individuals	515 individuals	SUCLG2 identified as both a determinator of CSF Aβ1-42 levels and an attenuator of cognitive decline in Alzheimer's disease.	Alzheimer's disease
(Jun *et al.*, 2017)	13,100 European ancestry cases, 13,220 European ancestry controls, 1,472 African American cases, 3,511 African American controls, 951 Japanese ancestry cases, 894 Japanese ancestry controls, 51 Israeli-Arab ancestry cases, 64 Israeli-Arab ancestry controls	5,813 European ancestry cases, 20,474 European ancestry controls	Transethnic genome-wide scan identifies novel Alzheimer's disease loci.	Alzheimer's disease
(Mukherjee *et al.*, 2018)	Up to 510 European ancestry cases, 3,447 European ancestry controls	NA	Genetic data and cognitively defined late-onset Alzheimer's disease subgroups.	Alzheimer's disease
(Harold *et al.*, 2009)	3,941 European ancestry cases, 7,848 European ancestry controls	2,023 European ancestry cases, 2,340 European ancestry controls	Genome-wide association study identifies variants at CLU and PICALM	Alzheimer's disease

			associated with Alzheimer's disease.	
(J. C. Lambert *et al.*, 2013)	2,025 European ancestry cases, 5,328 European ancestry controls	7,913 European ancestry cases, 10,417 European ancestry controls	Genome-wide haplotype association study identifies the FRMD4A gene as a risk locus for Alzheimer's disease.	Alzheimer's disease
(Jansen *et al.*, 2019)	24,087 European ancestry late-onset Alzheimer's disease cases, 47,793 European ancestry individuals with family history of Alzheimer's disease, 383,378 European ancestry controls	NA	Genome-wide meta-analysis identifies new loci and functional pathways influencing Alzheimer's disease risk.	Alzheimer's disease
(Jun *et al.*, 2016)	7,184 cases, 26,968 controls	718 European ancestry cases, 1,699 European ancestry controls	A novel Alzheimer disease locus located near the gene encoding tau protein.	Alzheimer's disease
(Hollingworth *et al.*, 2011)	6,688 European ancestry cases, 13,685 European ancestry controls	13,182 European ancestry cases, 26,161 European ancestry controls	Common variants at ABCA7, MS4A6A/MS4A4E, EPHA1, CD33 and CD2AP are associated with Alzheimer's disease.	Alzheimer's disease
(Naj *et al.*, 2011)	8,309 European ancestry cases, 7,366 European ancestry controls	10,523 European ancestry cases, 28,231 European ancestry controls	Common variants at MS4A4/MS4A6E, CD2AP, CD33 and EPHA1 are associated with late-onset Alzheimer's disease.	Alzheimer's disease
(Jean Charles Lambert *et al.*, 2013)	17,008 European ancestry cases, 37,154 European ancestry controls	8,572 European ancestry cases, 11,312 European ancestry controls	Meta-analysis of 74,046 individuals identifies 11 new susceptibility loci for Alzheimer's disease.	Alzheimer's disease
(Chung *et al.*, 2012)	443 European ancestry cases	NA	Genomic determinants of motor and cognitive outcomes in Parkinson's disease.	Parkinson's disease
(Wallen *et al.*, 2018)	1,950 European ancestry cases	726 European ancestry cases	Plasticity-related gene 3 (LPPR1) and age at diagnosis of Parkinson disease.	Parkinson's disease

(Hu *et al.*, 2016)	250 Han Chinese ancestry cases, 250 Han Chinese ancestry controls	NA	A Pooling Genome-Wide Association Study Combining a Pathway Analysis for Typical Sporadic Parkinson's Disease in the Han Population of Chinese Mainland.	Parkinson's disease
(Pickrell *et al.*, 2016)	9,619 European ancestry cases, 324,522 European ancestry controls	NA	Detection and interpretation of shared genetic influences on 42 human traits.	Parkinson's disease
(Nalls *et al.*, 2011)	5,333 European ancestry cases, 12,019 European ancestry controls	7,053 cases, 9,007 controls	Imputation of sequence variants for identification of genetic risks for Parkinson's disease: a meta-analysis of genome-wide association studies.	Parkinson's disease
(Hamza *et al.*, 2010)	2,000 European ancestry cases, 1,986 European ancestry controls	Up to 1,447 cases, 1,468 controls	Common genetic variation in the HLA region is associated with late-onset sporadic Parkinson's disease.	Parkinson's disease
(Maraganore *et al.*, 2005)	381 European ancestry cases, 363 European ancestry controls, 62 cases,79 controls, 1 Asian ancestry control from 443 sibships	269 European ancestry cases, 272 European ancestry controls, 62 cases, 60 controls, 1 Asian ancestry case	High-resolution whole-genome association study of Parkinson disease.	Parkinson's disease
(Fung *et al.*, 2006)	267 European ancestry cases, 270 European ancestry controls	NA	Genome-wide genotyping in Parkinson's disease and neurologically normal controls: first stage analysis and public release of data.	Parkinson's disease
(Satake *et al.*, 2009)	988 Japanese ancestry cases, 2,521 Japanese ancestry controls	933 Japanese ancestry cases, 15,753 Japanese ancestry controls	Genome-wide association study identifies common variants at four loci as genetic risk factors for Parkinson's disease.	Parkinson's disease
(Pankratz *et al.*, 2009)	1,119 European ancestry cases,	NA	Genomewide association study for susceptibility genes	Parkinson's disease

	1,127 European ancestry controls		contributing to familial Parkinson disease.	
(Edwards *et al.*, 2010)	1,752 European ancestry cases, 1,745 European ancestry controls	NA	Genome-wide association study confirms SNPs in SNCA and the MAPT region as common risk factors for Parkinson disease.	Parkinson's disease
(Do *et al.*, 2011)	3,426 European ancestry cases, 29,624 European ancestry controls	NA	Web-based genome-wide association study identifies two novel loci and a substantial genetic component for Parkinson's disease.	Parkinson's disease
(Liu *et al.*, 2011)	268 Ashkenazi Jewish cases, 178 Ashkenazi Jewish controls	1,782 European ancestry cases, 1,658 European ancestry controls	Genome-wide association study identifies candidate genes for Parkinson's disease in an Ashkenazi Jewish population.	Parkinson's disease
(Hill-Burns *et al.*, 2014)	1,565 European ancestry sproadic Parkinson's disease cases, 435 European ancestry familial Parkinson's disease cases, 1,986 European ancestry controls	1,528 European ancestry sporadic Parkinson's disease cases, 707 European ancestry familial Parkinson's disease cases, 796 European ancestry controls	Identification of a novel Parkinson's disease locus via stratified genome-wide association study.	Parkinson's disease
(Davis *et al.*, 2013)	31 Amish cases, 767 Amish controls	NA	Parkinson disease loci in the mid-western Amish.	Parkinson's disease
(Biernacka *et al.*, 2016)	364 cases, 364 sibling controls	NA	Genome-wide gene-environment interaction analysis of pesticide exposure and risk of Parkinson's disease.	Parkinson's disease
(Chang *et al.*, 2017)	20,184 European ancestry cases, 397,324 European ancestry controls	5,851 European ancestry cases, 5,866 European ancestry controls	A meta-analysis of genome-wide association studies identifies 17 new Parkinson's disease risk loci.	Parkinson's disease

(Hill-Burns *et al.*, 2016)	431 European ancestry cases	737 European ancestry cases	Identification of genetic modifiers of age-at-onset for familial Parkinson's disease.	Parkinson's disease
(Nalls *et al.*, 2011)	5,333 European ancestry cases, 12,019 European ancestry controls	7,053 cases, 9,007 controls	Imputation of sequence variants for identification of genetic risks for Parkinson's disease: a meta-analysis of genome-wide association studies.	Parkinson's disease
(Saad *et al.*, 2011)	1,039 European ancestry cases, 1,984 European ancestry controls	3,232 European ancestry cases, 7,064 European ancestry controls	Genome-wide association study confirms BST1 and suggests a locus on 12q24 as the risk loci for Parkinson's disease in the European population.	Parkinson's disease
(Hamza *et al.*, 2010)	2,000 European ancestry cases, 1,986 European ancestry controls	Up to 1,447 cases, 1,468 controls	Common genetic variation in the HLA region is associated with late-onset sporadic Parkinson's disease.	Parkinson's disease
(Pankratz *et al.*, 2012)	4,238 European ancestry cases, 4,239 European ancestry controls	3,738 European ancestry cases, 2,111 European ancestry controls	Meta-analysis of Parkinson's disease: identification of a novel locus, RIT2.	Parkinson's disease
(Latourelle *et al.*, 2009)	857 European ancestry familial cases, 440 idiopathic cases	747 European ancestry idiopathic cases	Genomewide association study for onset age in Parkinson disease.	Parkinson's disease
(Vacic *et al.*, 2014)	1,130 Ashkenazi Jewish cases, 2,611 Ashkenazi Jewish controls	306 Ashkenazi Jewish cases, 2,583 Ashkenazi Jewish controls	Genome-wide mapping of IBD segments in an Ashkenazi PD cohort identifies associated haplotypes.	Parkinson's disease
(Foo *et al.*, 2017)	779 Han Chinese ancestry cases, 13,227 Han Chinese ancestry controls	5,125 Asian ancestry cases, 17,604 Asian ancestry controls	Genome-wide association study of Parkinson's disease in East Asians.	Parkinson's disease
(Beecham *et al.*, 2015)	484 European ancestry cases, 1,145 European ancestry controls	NA	PARK10 is a major locus for sporadic neuropathologically	Parkinson's disease

			confirmed Parkinson disease.	
(Simón-Sánchez *et al.*, 2009)	1,713 European ancestry cases, 3,978 European ancestry controls	3,361 European ancestry cases, 4,573 European ancestry controls	Genome-wide association study reveals genetic risk underlying Parkinson's disease.	Parkinson's disease
(Nalls *et al.*, 2019)	15,056 European ancestry cases, 18,618 European ancestry proxy cases, 449,056 European ancestry controls	22,632 European ancestry cases, 968,735 European ancestry controls	Identification of novel risk loci, causal insights, and heritable risk for Parkinson's disease: a meta-analysis of genome-wide association studies.	Parkinson's disease
(Blauwendraat *et al.*, 2020)	1,588 European ancestry cases, 7,584 European ancestry controls	up to 1,194 European ancestry cases, up to 13,901 European ancestry controls	Genetic modifiers of risk and age at onset in GBA associated Parkinson's disease and Lewy body dementia.	Parkinson's disease
(Spencer *et al.*, 2011)	1,705 European ancestry cases, 5,175 European ancestry controls	1,039 European ancestry cases, 1,984 European ancestry controls	Dissection of the genetics of Parkinson's disease identifies an additional association 5' of SNCA and multiple associated haplotypes at 17q21.	Parkinson's disease
(Lill *et al.*, 2012)	2,197 cases, 2,061 controls	Up to 98,080 European and Asian ancestry individuals	Comprehensive research synopsis and systematic meta-analyses in Parkinson's disease genetics: The PDGene database.	Parkinson's disease
(Blauwendraat *et al.*, 2019)	17,996 cases	10,572 cases	Parkinson's disease age at onset genome-wide association study: Defining heritability, genetic loci, and α-synuclein mechanisms.	Parkinson's disease
(Nalls *et al.*, 2014)	13,708 European ancestry cases, 95,282 European ancestry controls	5,353 European ancestry cases, 5,551 European ancestry controls	Large-scale meta-analysis of genome-wide association data identifies six new risk loci for Parkinson's disease.	Parkinson's disease

(Bandres-Ciga *et al.*, 2019)	3,997 Spanish ancestry individuals	NA	The Genetic Architecture of Parkinson Disease in Spain: Characterizing Population-Specific Risk, Differential Haplotype Structures, and Providing Etiologic Insight.	Parkinson's disease
(Pottier *et al.*, 2018)	184 European ancestry cases, 198 European ancestry controls	NA	Potential genetic modifiers of disease risk and age at onset in patients with frontotemporal lobar degeneration and GRN mutations: a genome-wide association study.	Parkinson's disease
(Chao *et al.*, 2018)	374 Venezuelan ancestry individuals, 4,061 European ancestry individuals	NA	Population-specific genetic modification of Huntington's disease in Venezuela.	Huntington's disease
(Moss *et al.*, 2017)	1,989 European and unknown ancestry mutation carriers	NA	Identification of genetic variants associated with Huntington's disease progression: a genome-wide association study.	Huntington's disease
(Cronin *et al.*, 2009)	958 European ancestry cases, 932 European ancestry controls	309 European ancestry cases, 404 European ancestry controls	Screening for replication of genome-wide SNP associations in sporadic ALS.	Amyotrophic lateral sclerosis
(Chen *et al.*, 2016)	94 Taiwanese Han ancestry cases, 376 Taiwanese Han ancestry controls	NA	A genome-wide association study on amyotrophic lateral sclerosis in the Taiwanese Han population.	Amyotrophic lateral sclerosis
(McLaughlin *et al.*, 2015)	25 European ancestry C9orf72-positive cases, 1,179 European ancestry controls	NA	Second-generation Irish genome-wide association study for amyotrophic lateral sclerosis.	Amyotrophic lateral sclerosis
(Schymick *et al.*, 2007)	276 European ancestry cases, 271 European ancestry controls	NA	Genome-wide genotyping in amyotrophic lateral sclerosis and neurologically normal	Amyotrophic lateral sclerosis

			controls: first stage analysis and public release of data.	
(Cronin *et al.*, 2008)	221 Genetically Homogenous Irish cases, 211 Genetically Homogenous Irish controls	737 European ancestry cases, 721 European ancestry controls	A genome-wide association study of sporadic ALS in a homogenous Irish population.	Amyotrophic lateral sclerosis
(van Es *et al.*, 2007)	461 European ancestry cases, 450 European ancestry controls	876 European ancestry cases, 906 European ancestry controls	ITPR2 as a susceptibility gene in sporadic amyotrophic lateral sclerosis: a genome-wide association study.	Amyotrophic lateral sclerosis
(Laaksovirta *et al.*, 2010)	405 European ancestry cases, 497 European ancestry controls	NA	Chromosome 9p21 in amyotrophic lateral sclerosis in Finland: a genome-wide association study.	Amyotrophic lateral sclerosis
(Kwee *et al.*, 2012)	Up to 639 European ancestry cases, 6,257 European ancestry controls	Up to 183 European ancestry cases, 961 European ancestry controls	A high-density genome-wide association screen of sporadic ALS in US veterans.	Amyotrophic lateral sclerosis
(Van Es *et al.*, 2008)	737 European ancestry cases, 721 European ancestry controls	1,030 European ancestry cases, 1,195 European ancestry controls	Genetic variation in DPP6 is associated with susceptibility to amyotrophic lateral sclerosis.	Amyotrophic lateral sclerosis
(Wei *et al.*, 2019)	666 Han Chinese ancestry cases, 3,988 Han Chinese ancestry controls	up to 884 Han Chinese ancestry cases, up to 5,329 Han Chinese ancestry controls	Identification of TYW3/CRYZ and FGD4 as susceptibility genes for amyotrophic lateral sclerosis.	Amyotrophic lateral sclerosis
(Dekker *et al.*, 2019)	4,244 European ancestry cases, 3,106 European ancestry controls	NA	Exome array analysis of rare and low frequency variants in amyotrophic lateral sclerosis.	Amyotrophic lateral sclerosis
(Shatunov *et al.*, 2010)	4,857 European ancestry cases, 8,987 European ancestry controls	NA	Chromosome 9p21 in sporadic amyotrophic lateral sclerosis in the UK and seven other countries: a genome-wide association study.	Amyotrophic lateral sclerosis

(Van Es *et al.*, 2009)	2,323 European ancestry cases, 9,013 European ancestry controls	2,532 European ancestry cases, 5,940 European ancestry controls	Genome-wide association study identifies 19p13.3 (UNC13A) and 9p21.2 as susceptibility loci for sporadic amyotrophic lateral sclerosis.	Amyotrophic lateral sclerosis
(Langefeld, 2013)	4,243 European ancestry cases	NA	Age of onset of amyotrophic lateral sclerosis is modulated by a locus on 1p34.1.	Amyotrophic lateral sclerosis
(Deng *et al.*, 2013)	506 Han Chinese ancestry cases, 1,859 Han Chinese ancestry controls	706 Han Chinese ancestry cases, 1,777 Han Chinese ancestry controls	Genome-wide association analyses in Han Chinese identify two new susceptibility loci for amyotrophic lateral sclerosis.	Amyotrophic lateral sclerosis
(Goris *et al.*, 2014)	4,088 Multiple sclerosis cases, 3,762 Amyotrophic lateralsclerosis cases, 12,030 controls	NA	No evidence for shared genetic basis of common variants in multiple sclerosis and amyotrophic lateral sclerosis.	Amyotrophic lateral sclerosis
(Diekstra *et al.*, 2014)	4,377 European ancestry ALS cases, 435 European ancestry FTD cases, 14,431 European ancestry controls	4,056 European ancestry ALS cases, 3,958 European ancestry controls	C9orf72 and UNC13A are shared risk loci for amyotrophic lateral sclerosis and frontotemporal dementia: a genome-wide meta-analysis.	Amyotrophic lateral sclerosis
(Landers *et al.*, 2009)	1,821 cases, 2,258 controls	538 cases, 556 controls	Reduced expression of the Kinesin-Associated Protein 3 (KIFAP3) gene increases survival in sporadic amyotrophic lateral sclerosis.	Amyotrophic lateral sclerosis
(Van Rheenen *et al.*, 2016)	12,577 European ancestry cases, 23,475 European ancestry controls	2,579 European ancestry cases, 2,767 European ancestry controls	Genome-wide association analyses identify new risk variants and the genetic architecture of amyotrophic lateral sclerosis.	Amyotrophic lateral sclerosis
(Nicolas *et al.*, 2018)	20,806 European ancestry cases, 59,804 European ancestry controls	4,159 cases, 18,650 controls	Genome-wide Analyses Identify KIF5A as a Novel ALS Gene.	Amyotrophic lateral sclerosis

Table S2. Overview of studies used for gathering transcriptomic data—Transcriptome study information. AD = Alzheimer's disease, PD = Parkinson's disease, (s)ALS = (sporadic) amyotrophic lateral sclerosis, HD = Huntington's disease, ctrl = control, CSF = cerebrospinal fluid, BA = Brodman Area.

Study	Participants Per Experiment	Participants Total for Disease	Tissue
(Berchtold *et al.*, 2013)	26 AD, 33 old ctrl, 22 young ctrl	26 AD (13 severe, 9 moderate, 2 mild), 55 ctrl	Entorhinal Cortex, Hippocampus, Postcentral Gyrus, Superior Frontal Cortex
(Hokama *et al.*, 2014)	FC: 15 AD, 18 ctrl; Temporal Cortex: 10 AD, 19 ctrl; Hippocampus: 7 AD, 10 ctrl	32 AD (severe), 47 ctrl	Frontal Cortex, Temporal Cortex, Hippocampus
(Blalock *et al.*, 2004)	22 AD, 9 ctrl	22 AD (7 severe, 8 moderate, 7 mild), 9 ctrl	Hippocampus
(Blalock *et al.*, 2011)	22 AD (7 incipient, 8 moderate, 7 severe), 8 ctrl	22 AD (7 severe, 8 moderate, 7 mild), 8 ctrl	CA1 hippocampal gray matter
(Liang *et al.*, 2007)	EC: 10 AD, 13 ctrl; HIP: 10 AD, 13 ctrl; MTG: 16 AD, 12 ctrl; PC: 9 AD, 13 ctrl; SFG: 23 AD, 11 ctrl; VCX: 19 AD, 12 ctrl	24 severe AD, 10 moderate AD, 14 ctrl	EC=Entorhinal Cortex; HIP=Hippocampus; MTG=Medial temporal gyrus; PC=Posterior Singulate; SFG=Superior Frontal Gyrus; VCX=Primary visual cortex
(Magistri *et al.*, 2015)	4 LOAD, 4 ctrl	4 AD (severe), 4 ctrl	Hippocampus
(Dunckley *et al.*, 2006)	19 AD, 14 ctrl	19 AD (moderate), 14 ctrl	Entorhinal cortex
(Scheckel *et al.*, 2016)	9 AD, 8 ctrl	9 AD (severe), 8 ctrl	Brain
(Meyer *et al.*, 2019)	5 AD,5 ctrl	5 AD, 5 ctrl	iPSC induced neurons
(Mathys *et al.*, 2019)	24 AD, 24 ctrl	24 moderate AD (high ß-amyloid),	80660 sc transcriptomes from prefrontal cortex, 6 different

		24 mild ctrl (no ß-amyloid)	celltypes: astrocytes, exitatory neurons, inhibitory neurons, microglia, oligodendrocyte progenitor cells, oligodendrocytes
(Stopa *et al.*, 2018)	7 AD, 3 HD, 6 ctrl	6 severe AD, 1 moderate AD, 6 ctrl	choroid plexus
(Simunovic *et al.*, 2009)	10 PD, 9 ctrl	10 PD, 9 ctrl	neurons of Substantia nigra
(Elstner *et al.*, 2011)	11 PD, 11 old ctrl, 8 young ctrl	11 PD, 19 ctrl	neurons of Substantia nigra
(Riley *et al.*, 2014)	16 PD-20 ctrl (FC), 17 PD-17 ctrl (striatum), 16 PD-14 ctrl (SN)	24 PD, 22 ctrl	Cortex, Striatum, Substantia nigra
(Dijkstra *et al.*, 2015)	9 PD, 8 ctrl	9 PD, 8 ctrl	Substantia nigra
(Dumitriu *et al.*, 2012)	27 PD, 26 ctrl	27 PD, 26 ctrl	prefrontal cortex (BA9)
(Dumitriu *et al.*, 2016)	29 PD, 44 ctrl	29 PD, 44 ctrl	prefrontal cortex (BA9)
(Scherzer *et al.*, 2007)	50 PD, 22 ctrl	50 mild PD, 22 ctrl	whole blood
(Lesnick *et al.*, 2007)	16 PD, 9 ctrl	16 PD, 9 ctrl	Substantia nigra
(Zhang *et al.*, 2005)	15 PD, 15 ctrl (each tissue)	15 PD, 15 ctrl	Prefrontal cortex (BA9), substantia nigra
**GSE20333_Edna_2010_PD**	6 PD, 6 ctrl	6 PD, 6 ctrl	Substantia nigra
(Calligaris *et al.*, 2015)	40 PD, 20 ctrl	40 mild PD, 20 ctrl	Blood
(Ring *et al.*, 2015)	16 HD, 16 ctrl	16 severe HD, 16 ctrl	Transcriptomic analysis of HD iPSCs and HD NSCs compared to isogenic controls using RNA-Seq.
(Świtońska *et al.*, 2019)	2HD, 2 ctrl	2 severe HD, 2 ctrl	HD and control iPSC lines
(Al-Dalahmah *et al.*, 2020)	6 HD Grad III/IV, 6 ctrl	6 severe HD, 6 ctrl	astrocytes of singulate cortex
(Labadorf *et al.*, 2015)	20 HD, 49 ctrl, male	20 severe HD, 49 ctrl	prefrontal cortex, Brodman Area 9
(Lin *et al.*, 2016)	7 HD, 7 ctrl	7 severe HD, 7 ctrl	motor cortex
(Feyeux *et al.*, 2012)	6 HD, 4 ctrl	6 mild HD, 4 ctrl	embryonic stem cells

(Lim, Salazar, *et al.*, 2017)	6 HD, 4 ctrl	6 HD, 4 ctrl	iPSC derived neural cells
(Lim, Quan, *et al.*, 2017)	4 HD, 2 ctrl	4 HD, 2 ctrl	iPSC induced brain microvascular endothelial cells
(Mehta *et al.*, 2018)	3 HD, 3 ctrl	3 severe HD, 3 ctrl	iPSC derived cortical neurons
(Stopa *et al.*, 2018)	7 AD, 3 HD, 6 ctrl	3 HD, 6 ctrl	choroid plexus
(Cox *et al.*, 2010)	3 ALS, 7 ctrl	3 ALS, 7 ctrl	cervical spinal cord
(Otake, Kamiguchi and Hirozane, 2019)	4 ALS, 4 ctrl	4 ALS, 4 ctrl	exosomale mRNA in CSF
(Gagliardi *et al.*, 2018)	11 sALS, je 2 fALS(FUS,TARDBP,SOD1), 3 ctrl	11 sALS, 3 ctrl	blood_monocytes
(Swindell *et al.*, 2019)	397 ALS, 645 ctrl	397 moderate ALS, 645 ctrl	blood
(Prudencio *et al.*, 2015)	19 ALS (10 sALS, 9 c9ALS), 9 ctrl	19 severe ALS, 9 ctrl	Cerebellum (BA9/44), Frontal cortex (BA4)
(Raman *et al.*, 2015)	6 sALS, 6 ctrl	6 sALS, 6 ctrl	Fibroblasts from skin cells
(Kapeli *et al.*, 2016)	13 sALS, 9 control	13 sALS, 9 control	human stem cell-derived motor neurons
(Dols-Icardo *et al.*, 2020)	11 ALS, 8 ctrl	11 ALS, 8 ctrl	motor cortex, BA 4

Table S3. Overview of studies used for gathering proteomic data— Proteome study information. AD = Alzheimer's disease, PD = Parkinson's disease, ALS = amyotrophic lateral sclerosis, HD = Huntington's disease, ctrl = control, CSF = cerebrospinal fluid, BA = Brodman Area.

Study	Participants Per Experiment	Participants Total for Disease	Tissue
(Johnson *et al.*, 2020)	268A D, 104 asymptomatic AD, 99 ctrl	268A D, 104 asymptomatic AD, 99 ctrl	dorsolateral prefrontal cortex
(Bader *et al.*, 2020)	87 AD, 67 ctrl	87 AD, 67 ctrl	CSF
(Zhang *et al.*, 2018)	8 AD, 8 ctrl	8 severe AD, 8 ctrl	frontal cortex
(Seyfried *et al.*, 2017)	Emory: each 8 AD, PD, ALS, ctrl;<br>BLSA: 20 AD (BA7), 20 AD (BA9), 15 ctrl (BA7), 13 ctrl (BA9)	28 severe AD, 15 moderate, 22 mild ctrl	Emory: prefrontal cortex;<br>BLSA: prefrontal cortex (BA9), Precuneus (BA7)
(Higginbotham, Dammer, *et al.*, 2019)	6 severe AD, 6 moderate AD, 6 mild ctrl	6 severe AD, 6 moderate AD, 6 mild ctrl	middle frontal gyrus BA 8, BA 9
(Higginbotham, Ping, *et al.*, 2019)	CSF1: 20 AD, 20 ctrl; Brain1: 10 AD, 10 ctrl; Brain2: 9 AD, 10 ctrl; CSF2: 33 AD, 32 ctrl	72 severe AD, 72 mild ctrl	dorsolateral prefrontal cortex

(Wingo *et al.*, 2020)	383 AD, 375 ctrl	122 severe AD, 101 moderate AD, 160 mild ctrl	dorsolateral prefrontal cortex (BA9)
(Lachén-Montes *et al.*, 2017)	each 5 AD (3 states), 5 ctrl	5 severe AD, 5 moderate AD, 5 mild AD, 5 ctrl	olfactory bulb
(Hondius *et al.*, 2016)	35 AD, 5 ctrl	7 severe AD, 7 moderate AD, 7 mild AD, 5 ctrl	hippocampus: CA1 + subiculum
(Dumitriu *et al.*, 2016)	12 PD, 12 ctrl	12 PD, 12 ctrl	prefrontal cortex BA9
(Rotunno *et al.*, 2020)	Cohort 1: 53 PD, 72 ctrl<br>Cohort 2: 28 PD, 43 ctrl	81 moderate PD, 115 ctrl	CSF
(Riley *et al.*, 2014)	3 PD, 3 ctrl (striatum), 5 PD, 5 ctrl (Cortex)	8 PD, 8 ctrl	Striatum, cortex
(Seyfried *et al.*, 2017)	8 PD, 8 ctrl	8 PD, 8 ctrl	Prefrontal cortex
(Lachén-Montes *et al.*, 2019)	21 PD, 8 ctrl	21 PD, 8 ctrl	olfactory bulb
(Higginbotham, Ping, *et al.*, 2019)	10 PD, 10 ctrl	10 PD, 10 ctrl	dorsolateral prefrontal cortex
(van Dijk *et al.*, 2012)	6 PD, 6 ctrl	6 PD, 6 ctrl	locus ceruleus
(Umoh *et al.*, 2018)	19 ALS, 10 ctrl	19 ALS, 10 ctrl	frontal cortex
(Oeckl *et al.*, 2020)	26 ALS, 16 ctrl (CSF), 8ALS, 7ctrl (spinal cord tissue)	34 severe ALS, 21 asympt. ctrl	CSF, spinal cord tissue
(Iridoy *et al.*, 2019)	9 ALS, 8 ctrl	9 ALS, 8 ctrl	anterior horn of spinal cord
(Collins *et al.*, 2015)	90 sALS, 80 ctrl, pooled to 9 sALS, 8 ctrl	90 sALS, 80 ctrl	CSF
(Varghese *et al.*, 2013)	10 ALS, 10 ctrl	10 ALS, 10 ctrl	CSF
(Ratovitski *et al.*, 2016)	12 HD, 12 ctrl	12 HD, 12 ctrl	cortex
(Fang *et al.*, 2009)	10 earlyHD, 10 midHD, 10 ctrl	10 earlyHD, 10 midHD, 10 ctrl	CSF
(McQuade *et al.*, 2014)	hESC: 3 HD, 3 ctrl<br>NSC: 4 HD, 4 ctrl	7 adult-onset HD, 7 ctrl	hESC, NSC
