# Supplementary figures and images for: Common Factors in Neurodegeneration: A Meta-Study Revealing Shared Patterns on a Multi-Omics Scale
Source: Cells. 2020 Dec 8;9(12):2642. doi: 10.3390/cells9122642 (PMC7764447; doi:10.3390/cells9122642)

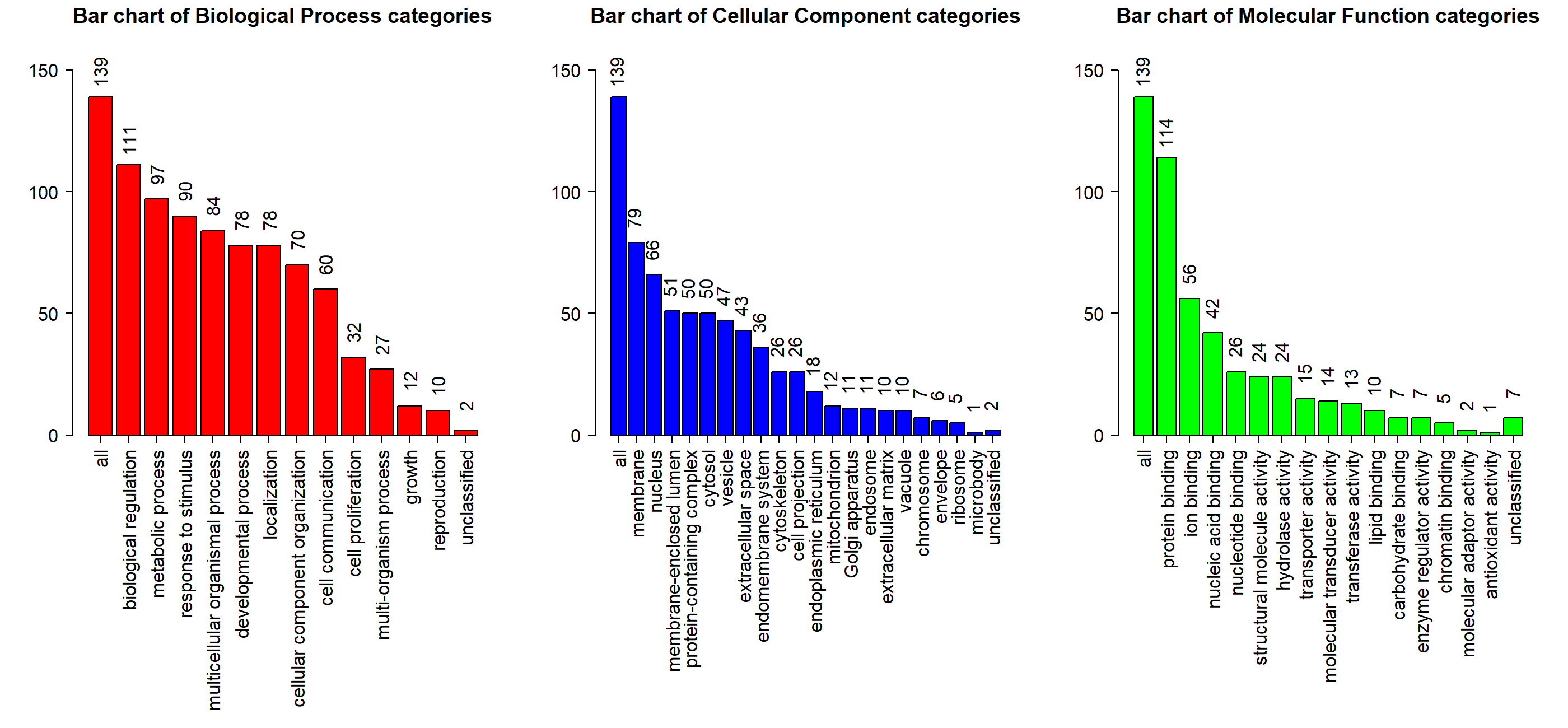

Supplement: Supplementary file 1 [file cells-09-02642-s001.zip › Supplementary/S5_GO-Term and Pathway Analyses/Project_AD_PD_HD_ALS_DEG/goslim_summary_AD_PD_HD_ALS_DEG.png]

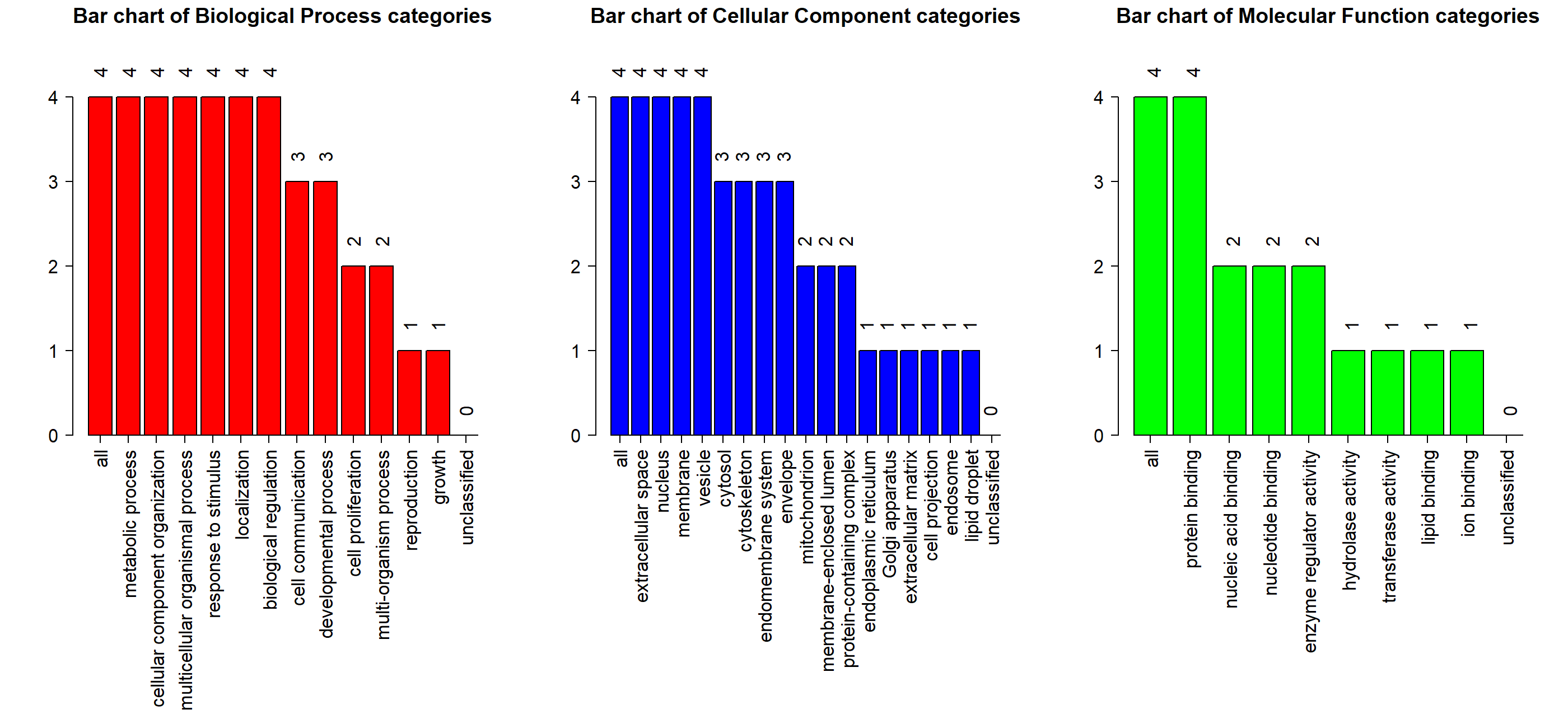

Supplement: Supplementary file 1 [file cells-09-02642-s001.zip › Supplementary/S5_GO-Term and Pathway Analyses/Project_AD_PD_HD_ALS_Prot/goslim_summary_AD_PD_HD_ALS_Prot.png]
